# Cell Area Characterisation of Fe-B-C Cast Alloys with Various Additions of Cr, V, W, Ti, Nb and Mo Measured by Optical Microscopy Method

**DOI:** 10.3390/ma19214709

**Published:** 2026-11-05

**Authors:** Lenka Křivánková, Rostislav Chotěborský, Barbora Kuřetová

**Affiliations:** 1 Department of Material Science and Manufacturing Technology, Faculty of Engineering, Czech University of Life Sciences Prague, Kamycka 129, 165 00 Prague, Czech Republic; krivankoval@tf.czu.cz (L.K.); choteborsky@tf.czu.cz (R.C.); 2 Department of Electrical Engineering and Automation, Faculty of Engineering, Czech University of Life Sciences Prague, Kamycka 129, 165 00 Prague, Czech Republic

**Keywords:** Fe–B alloys, microstructure measurement, image analysis, cell area distribution, boride morphology

## Abstract

**Highlights:**

- Full-area microscopy maps revealed core-to-surface matrix cell refinement in Fe–B–C alloys.
- The effect of the alloying elements Cr, V, W, Ti, Nb and Mo on cell area was evaluated.
- Boride morphology ranged from network-like to strongly fragmented at approximately 0.35 wt.% B and it depended on the accompanying alloying elements.

**Abstract:**

This study developed an automated workflow for spatially resolved quantitative characterisation of Fe–B–C alloy microstructures across sample cross-sections. Automated high-magnification optical microscopy and image reconstruction generated spatially registered maps exceeding 20 GB per sample, preserving microstructural detail and spatial position. The workflow quantified cell area and core-to-surface gradients, while the maps supported assessment of boride-network distribution and morphology. It was applied to 22 hypoeutectic alloys containing approximately 0.35 wt.% B, prepared under identical casting conditions, and modified with Cr, V, W, Ti, Nb, and Mo. Optical emission spectroscopy and scanning electron microscopy with energy-dispersive X-ray spectroscopy complemented full-area image analysis and statistical evaluation. After invalid measurements and geometrically unsuitable regions were excluded, 12,785 figures with valid cell area measurements were analysed. All alloys exhibited a decrease in cell area from the core towards the surface. Alloy-group differences were significant for mean and median cell area according to one-way analysis of variance (ANOVA; *p* < 0.001) and the Kruskal–Wallis test (*p* = 0.027 for both parameters), whereas the magnitude of the spatial gradient did not differ significantly among groups. The workflow provides a reproducible link between high-resolution microscopy and sample-scale evaluation of microstructural distributions, gradients, and homogeneity.

## 1. Introduction

The development of materials exhibiting high resistance to abrasive wear remains one of the leading research topics in materials engineering, particularly for components exposed to severe mechanical and tribological conditions. Extending service life provides significant economic and operational benefits in this area [1,2,3]. Among the materials developed for such applications, Fe–B alloys have attracted considerable attention because boron can significantly modify the microstructure and mechanical properties of iron-based alloys. At very low concentrations (typically 0.0005–0.003 wt.%), boron enhances the hardenability of steels by delaying diffusional phase transformations and promoting martensitic hardening [4,5]. However, this effect is highly sensitive to both boron concentration and metallurgical purity, and excessive boron additions may reduce the expected improvement in hardenability [5,6,7]. Once the boron content exceeds its solubility limit in austenite and ferrite (≤0.02 wt.% in γ-Fe and ≤0.0081 wt.% in α-Fe), the strengthening mechanism changes fundamentally [2]. Furthermore, the solubility of boron in iron may be modified by alloying additions such as Cr, Mo and V [8,9,10].

In high-boron alloys (typically ≥1 wt.% B), Fe_2_B borides replace conventional carbide phases and provide hardness levels exceeding those of typical carbides, making these alloys particularly suitable for abrasive wear applications [11,12,13,14]. However, despite these advantages, their performance under severe service conditions involving impact or dynamic loading is often limited by the intrinsic brittleness of the Fe_2_B phase [1,11,15]. During abrasive or impact wear, Fe_2_B particles are prone to cracking and subsequent spallation, reducing their ability to protect the comparatively softer metallic matrix and consequently decreasing the overall wear resistance, particularly under impact loading [8,15].

At boron concentrations above the solubility limit but below those typical of classical high-boron alloys (generally < 1 wt.% B), partial boride formation may occur without necessarily developing a fully continuous boride network [16,17]. Representative microstructures reported by Zhang et al. [18] showed that lower boron contents (0.5 wt.% B) are associated with discontinuous rod-like and polymorphic borides embedded within a continuous metallic matrix, whereas increasing boron concentration promotes the development of interconnected fishbone and network-like boride morphologies at boron contents of 1.0, 1.5, and 2.0 wt.% B.

In alloys with intermediate boron concentration, strengthening results from the combined effect of matrix hardening and the precipitation of discrete Fe_2_B particles, increasing hardness and improving abrasive wear resistance while preserving a continuous metallic matrix [2,11,19,20]. Compared with high-boron alloys, these materials exhibit a less continuous boride network, higher toughness, and a bainitic matrix containing retained austenite, making them more suitable for subsequent thermomechanical processing [18]. Furthermore, repeated hot forging of Fe–B cast steel containing 0.38–0.42 wt.% B promotes boride fragmentation and redistribution within the metallic matrix, while subsequent heat treatment leads to boride spheroidization and further improves toughness [21]. Despite these advantages, the resulting microstructure remains highly sensitive to the morphology, size, and spatial distribution of borides. Consequently, the mechanisms governing the balance between hardness and fracture resistance, particularly under dynamic or impact loading, are still not fully understood [8,14,20]. Therefore, intermediate-boron Fe–B alloys represent a promising but still insufficiently explored class of materials that may bridge the performance gap between conventional micro-alloyed steels and high-boron alloys.

In Fe–B–C alloys, alloying additions significantly influence phase equilibria, solidification pathways, and transformation kinetics, thereby modifying microstructural evolution and the resulting mechanical properties [1,13,15,22,23,24,25]. Although the beneficial effects of alloying elements on Fe–B alloys have been widely reported, their individual influence on the microstructure of alloys with intermediate boron contents has not yet been comprehensively understood.

Transition metals such as Cr, Mo, V, and W, as well as strong carbide- and boride-forming elements including Ti and Nb, are commonly employed to tailor the solidification behaviour and microstructural evolution of Fe–B–C alloys [24,26]. These alloying additions primarily influence boride morphology and microstructural evolution, thereby affecting the resulting mechanical properties of Fe–B–C alloys.

Chromium partially substitutes for Fe in Fe_2_B, forming (Fe,Cr)_2_B or related borocarbides, which enhances boride toughness. It also modifies eutectic morphology, promoting a more discontinuous boride network and thereby improving fracture resistance [22,23,26]. Jian et al. (2024) [1] reported that the addition of approximately 2 wt.% Cr to an Fe–3.0 wt.% B alloy improved abrasion resistance, whereas further increases in Cr content led to diminishing improvements. Furthermore, Cr has been reported to interact with V during solidification without fundamentally altering the overall phase constitution [8]. The combined addition of Cr and V effectively controls boride crystallization behaviour and suppresses the preferential directional growth of primary Fe_2_B, resulting in a finer and less interconnected boride network [8,9]. Recent studies further indicate that Cr incorporation at the atomic level may modify bonding characteristics within Fe_2_B, contributing to improved intrinsic toughness of the boride phase [10,27,28].

Besides chromium, molybdenum is another alloying element that significantly influences boride morphology and microstructural evolution in Fe–B–C alloys [1,9,24,29,30]. Jian et al. (2024) [1] demonstrated that Mo promotes the formation of Mo-rich borides, such as M_3_B_2_ and FeMo_2_B_2_, while modifying the morphology of the primary M_2_B (Fe_2_B-type) phase. The formation of these Mo-containing phases disrupts the continuity of the boride network and alters solidification pathways, leading to changes in fracture behaviour [1,9]. Chen et al. [24] investigated the effect of Mo additions ranging from 0 to 2.86 wt.% in high-boron Fe–B cast steel and reported that increasing Mo content progressively refined the originally continuous fishbone-like M_2_B boride network into shorter and more isolated boride morphologies. This microstructural modification reduced crack propagation susceptibility and improved toughness while maintaining high hardness and wear resistance [24].

Vanadium additions have similarly been shown to influence the microstructure of Fe–B alloys by refining primary Fe_2_B and promoting the precipitation of hard VB borides [9,15]. These microstructural changes contribute to reduced heterogeneity and improved mechanical performance. In addition, W has been shown to influence solidification kinetics and boride growth, reducing the formation of a continuous Fe_2_B network and promoting a finer boride morphology [25].

In boron-rich Fe–B–C alloys, grain size is a key descriptor of microstructural organization because it reflects solidification behaviour and governs the spatial arrangement of boride phases [11]. Variations in grain size influence boride distribution, network connectivity, and local stress concentrations, thereby affecting fracture behaviour and wear resistance [8,11,20]. Therefore, evaluation of both the average grain size and its spatial heterogeneity provides valuable insight into the structural stability and overall performance of these alloys [31].

Reliable characterization of grain size requires analysis of the entire cross-section rather than isolated or arbitrarily selected image fields [32]. Localized measurements may fail to capture spatial gradients and microstructural heterogeneity arising during solidification [33,34,35]. Consequently, full-area microstructural analysis provides a more representative and physically meaningful characterization of phase distribution, connectivity, and the resulting mechanical response in boride-containing systems [31,33,35,36].

Several approaches have been proposed for the characterization of microstructural heterogeneity. Network-based methods represent grains as interconnected nodes and quantify structural heterogeneity using graph-theoretical descriptors [35]. Another approach is to reconstruct large-scale microstructural images from multiple image acquisitions, enabling the assessment of spatial heterogeneity and structural variations across extended sample areas [37].

In our previous study (Křivánková et al. [38], 2025), a methodology for full-area evaluation of cell area in hypoeutectic Fe–B–C alloys was developed using large-area optical microscopy and automated image analysis. The development of Fe–B–C alloys for agricultural tools requires high wear resistance combined with sufficient hot workability for forging or hot pressing. Achieving these properties involves controlling the balance between the metallic matrix and hard boride phases, particularly their morphology and spatial distribution. Microstructural characterisation therefore represents an important step in selecting promising alloys for subsequent wear and field testing. The novelty of this study lies in the systematic evaluation of cell area and its spatial variation across the casting cross-section, enabling comparison of microstructural homogeneity among experimental alloys with different additions of Cr, V, W, Ti, Nb, and Mo. Building on this approach, the present study investigates the effects of Cr, V, W, Ti, Nb, and Mo on cell area and its spatial distribution in experimental Fe–B–C alloys containing approximately 0.35 wt.% B. The analysis focuses on cell area distributions, where cell area refers to the characteristic size of microstructural regions bounded by the boride network, radial cell area gradients, and overall microstructural heterogeneity across the casting cross-section. In addition, the morphology and network connectivity of the boride phase are examined to assess their relationship with alloy composition.

## 2. Materials and Methods

### 2.1. Design of Experimental Alloys

**Figure 1:**
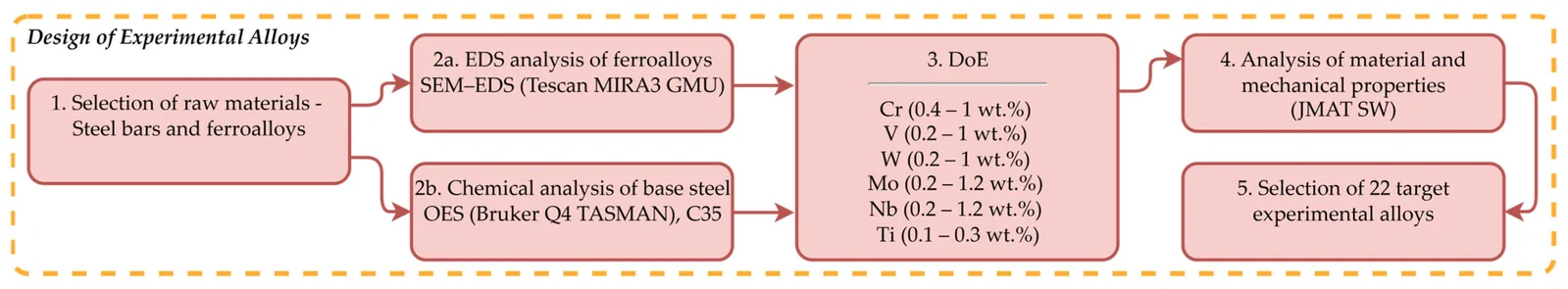
Flowchart of the alloy design procedure, including raw material selection, compositional definition, thermodynamic analysis, and final composition selection.

Prior to alloy preparation, the chemical compositions of the investigated alloys were designed following the workflow summarized in Figure 1. The design procedure comprised raw material selection, chemical characterization of the starting materials, Design of Experiments (DoE), thermodynamic simulations using JMatPro (version 13.2; Sente Software Ltd., Guildford, UK) [39], and final selection of the experimental alloy compositions. The individual steps are described in the following sections.

#### 2.1.1. Raw Material Selection and Chemical Composition Analysis

The alloys were prepared using the ferroalloys FeB, FeCr LC, FeW, FeV, FeTi, FeNb, and FeMo as sources of boron and alloying elements influencing boride formation, solidification behaviour, and matrix microstructure. Their pre-alloyed form ensured uniform dissolution in the melt and homogeneous incorporation into the liquid steel. C35 steel bars (30 mm in diameter) served as the base material.

Before designing the experimental alloys, the chemical compositions of the base steel and ferroalloys were determined as part of our previous study (Křivánková et al. [38], 2025) and are summarized in Table 1. The ferroalloys were analysed by energy-dispersive X-ray spectroscopy (EDS) using a Tescan MIRA3 GMU (TESCAN, Brno, Czech Republic) scanning electron microscope (SEM), whereas the chemical composition of the C35 steel was determined by optical emission spectroscopy (OES) using a Bruker Q4 TASMAN (Bruker AXS, Karlsruhe, Germany) spectrometer.

#### 2.1.2. Design of Experiments

A Design of Experiments (DoE) approach was adopted to design the experimental alloys. The alloy compositions were generated using JMatPro 13.2 software by varying the concentrations of Cr, V, W, Mo, Nb, and Ti, whereas the boron content was fixed at 0.4 wt.% through the addition of ferroboron (FeB). Chromium, introduced as low-carbon ferrochromium (FeCr LC), was investigated at two concentration levels (0.4 and 1.0 wt.%), representing the lower and upper limits of the studied compositional range. The alloy compositions were based on C35 steel and the selected ferroalloys (FeB, FeCr LC, FeW, FeV, FeTi, FeNb, and FeMo), while the investigated concentration ranges and discrete concentration levels are summarized in Table 2.

The concentration limits were established using preliminary thermodynamic and kinetic calculations in JMatPro to promote boride formation while suppressing competing phases, particularly carbides. The upper concentration limits were selected to minimize carbide formation and thus preserve sufficient carbon in the metallic matrix for subsequent heat treatment. The calculations included equilibrium phase diagrams (isopleths), temperature-dependent phase fraction analyses, continuous cooling transformation (CCT) and time–temperature–transformation (TTT) diagrams, Jominy hardenability curves, and tempering response predictions. Based on the calculated phase constitution and predicted microstructure, 72 alloy compositions were generated, from which 22 representative alloys were selected for experimental preparation. The selection was primarily based on minimizing undesirable carbide formation while ensuring the formation of borides and the desired characteristics of the metallic matrix.

#### 2.1.3. Target Experimental Alloy Compositions

Based on the simulation results, 20 alloys exhibiting suitable predicted thermodynamic and kinetic behaviour were selected for further experimental investigation. The chemical compositions of the selected experimental alloys are summarized in Table 3 under the column “Target Alloy Chemical Composition”, while the corresponding predicted microstructures and phase compositions after simulated quenching from the austenitisation temperature are presented in the subsequent columns.

Although the table contains 22 alloy entries, only 20 unique chemical compositions were designed. Two compositions were intentionally prepared in duplicate to assess the reproducibility of alloy preparation; therefore, samples E5 and E7 share identical nominal compositions, as do samples E6 and E8. Furthermore, the chemical compositions of samples E1–E8 correspond to those of samples K1–K8 reported in our previous study [38]. Table 3 also shows the theoretical phase composition obtained by the JMatPro “Solidification Properties” tool for all 22 experimental alloys. The column “Martensite (Phase 1)” indicates the total fraction of the matrix phase. The “Borides” column includes the combined fraction of Fe_3_B and Fe_2_B. Carbides M(C,N) and M_23_C_6_ were evaluated together. “Sum of Phases 2” represents the total fraction of borides and carbides.

### 2.2. Sample Preparation and Microstructural Analysis

**Figure 2:**
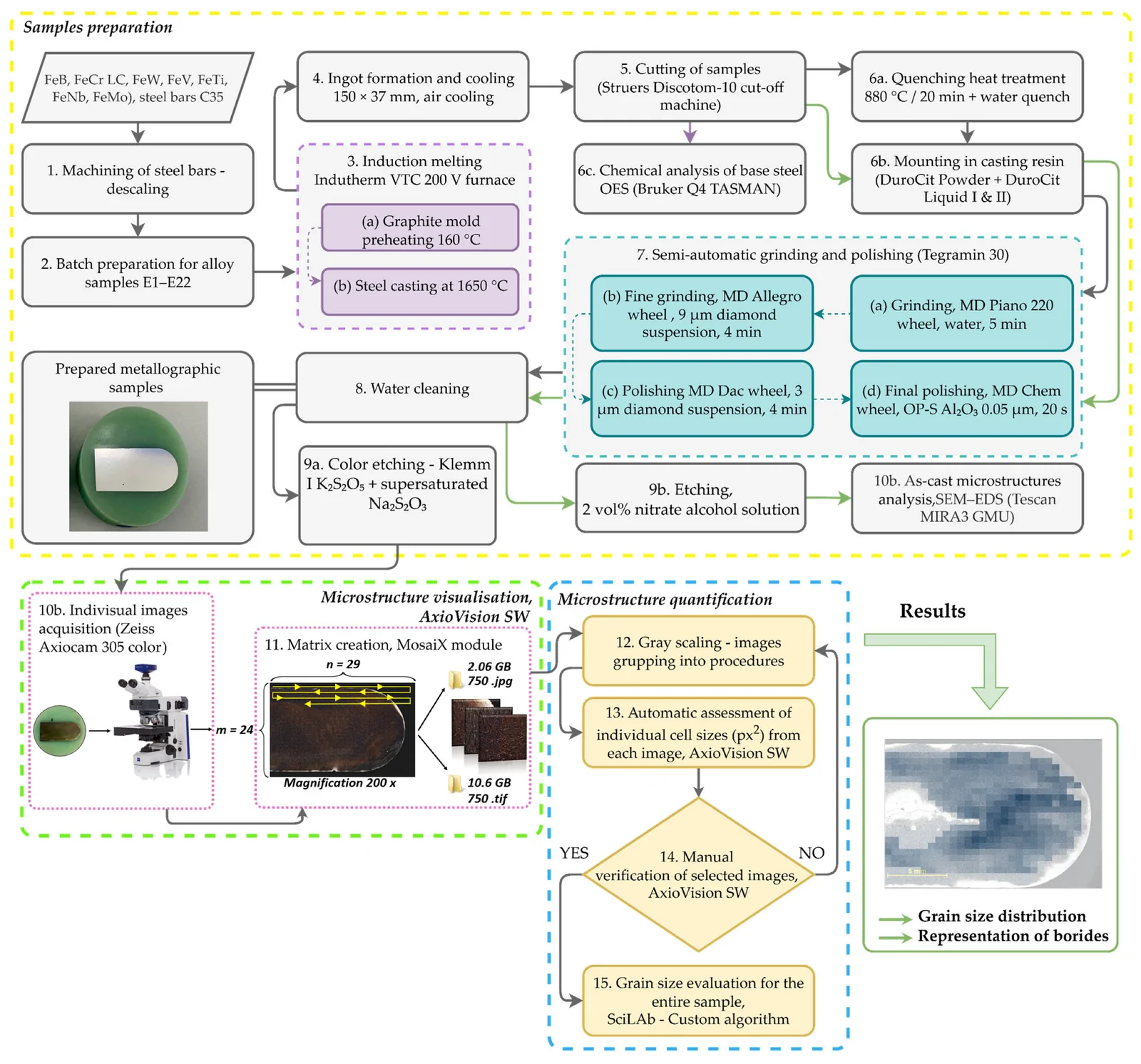
Schematic overview of the procedure applied for microstructural analysis, including sample preparation (highlighted in yellow), microstructural visualisation (highlighted in light green), and automated microstructural quantification (highlighted in blue). The resulting outputs consisted of microstructural maps providing cell area distribution and boride phase representation (highlighted in dark green).

Following alloy design, the experimental alloys were cast, metallographically prepared, and subjected to chemical and microstructural characterization. The complete experimental workflow is summarized in Figure 2 and described in the following sections.

#### 2.2.1. Metallographic Sample Preparation

The experimental alloys were prepared from C35 steel and the selected ferroalloys. Prior to melting, the C35 steel bars were machined to remove surface scale and sectioned into approximately 100 mm long cylindrical segments. The raw materials were then weighed according to the target compositions of alloys E1–E22; the corresponding charge compositions are summarized in Table 4.

**Figure 3:**
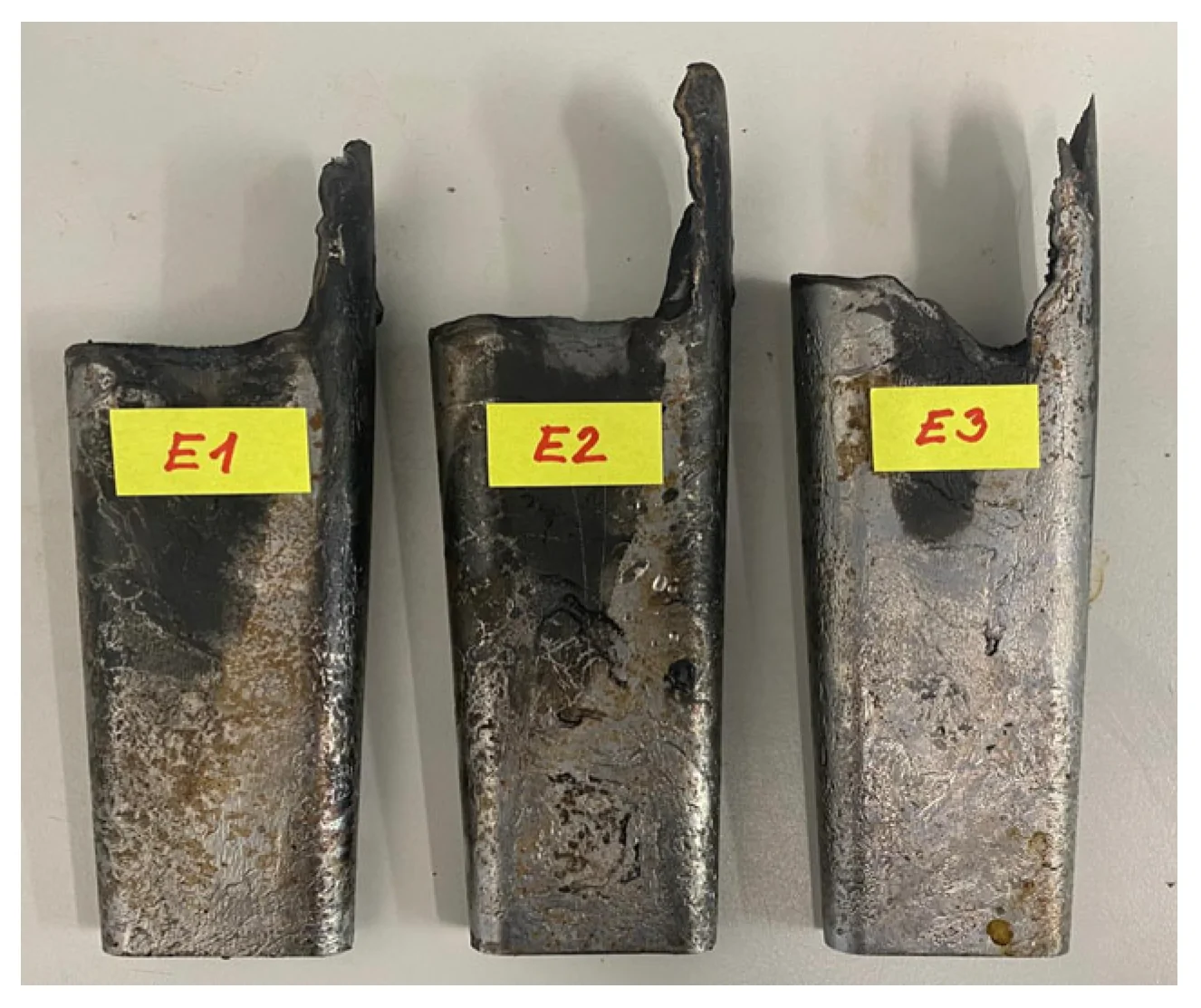
Final shape of the air-cooled castings, illustrated for the representative experimental alloys E1, E2, and E3.

The prepared charges were melted in a vacuum medium-frequency induction furnace (VTC 200 V; INDUTHERM Erwärmungsanlagen GmbH, Walzbachtal, Germany) and poured at 1650 °C into a graphite mould preheated to 160 °C. After solidification, the castings were removed from the mould and cooled in air at room temperature. Representative castings are shown in Figure 3.

The castings were subsequently sectioned and prepared for three analytical routes. For optical microstructural analysis, the samples were quenched, mounted in resin, ground, polished, cleaned, and etched with Klemm I reagent. This preparation procedure, established in our previous study [38], provides suitable contrast between the light boride phases and the dark blue or brown matrix. For SEM–EDS analysis, the samples were mounted, ground, polished, cleaned, and etched with a 2% nitric acid–alcohol solution. The bulk chemical composition of the cast alloys was additionally evaluated by OES directly after sectioning.

#### 2.2.2. Microstructure Visualisation and Microstructural Quantification

After metallographic preparation, optical micrographs were acquired using AxioVision SE64 software (version 4.9.1; Carl Zeiss Microscopy GmbH, Jena, Germany) and assembled into continuous image matrices covering the entire sample cross-section.

For automated microstructural quantification, the acquired images were grouped according to their mean greyscale intensity to account for variations in surface condition and illumination that could affect cell-boundary detection. Each greyscale group, hereafter referred to as a procedure, involved specific parameter settings within the software to optimise cell boundary detection and ensure consistent automatic extraction of cell area data.

The defined procedures were manually verified using images at the limiting greyscale values of each group and adjusted when necessary. After validation, the image-analysis algorithm developed in our previous study [38] was applied to the entire sample area to determine the cell area distribution and boride phase representation. Image frames located at the sample boundaries, containing predominantly mounting resin or insufficiently resolved microstructural regions, were excluded from quantitative evaluation. The identification of unsuitable frames was based on mean greyscale intensity classification and subsequent visual verification, following the methodology established in our previous study [38]. This procedure was used to minimise the influence of non-representative image regions on the automated cell area measurements.

### 2.3. Statistical Analysis

#### 2.3.1. Cell Area Characterisation and Gradient Analysis

The gradient of the matrix cell area across the casting cross-section was determined from the dependence between the average matrix cell area *d* and the distance from the casting centre *x*. Measurements were performed at several systematically defined positions along the radial direction from the core towards the surface of the casting.

For each measurement position, the arithmetic mean, median, standard deviation, and number of valid measurements were calculated. Summary statistics were also determined for each sample, including the overall mean, median, minimum, maximum, and standard deviation of all valid cell area measurements.

For each position, the average matrix cell area was determined from metallographic micrographs using image analysis. The dependence of cell area on distance from the casting centre was initially evaluated by linear regression of all individual cell area measurements against measurement position. Because this approach was strongly affected by local variability and by the unequal number of measurements at individual positions, the primary analysis was based on the mean cell area calculated for each measurement position, with each position representing a single point in the regression. The relationship between cell area and distance from the casting centre was described using linear regression according to Equation (1).

*d*(*x*)* = ax *+* b* (1)

where *d*(*x*) is the average cell area at distance *x*, *a* is the cell area gradient, and *b* represents the intercept corresponding to the estimated cell area in the central region of the casting.

The regression coefficients were determined using the least squares method. The gradient coefficient *a* represents the rate of change of cell area with increasing distance from the casting centre and was used as a quantitative parameter describing the microstructural gradient. The statistical significance of the regression slope was assessed using a two-sided test of the null hypothesis that the slope was equal to zero. The test was based on the standard error of the slope and Student’s (t)-distribution with (*n* − 2) degrees of freedom, where (*n*) represented the number of measurement positions included in the regression. As a robust complementary estimator, the Theil-Sen slope was also calculated because of its lower sensitivity to extreme observations. The calculated gradient values were subsequently used as response variables in statistical analysis.

#### 2.3.2. Relationship Between Chemical Composition and Cell Area Parameters

The relationships between chemical composition and cell area parameters were evaluated for Cr, V, W, Ti, Nb, and Mo. Values below the limit of detection (LOD) were replaced by one-half of the corresponding LOD in the primary analysis. Sensitivity analyses were additionally performed by replacing these values with zero and with the full LOD.

The relationships between the concentrations of individual alloying elements and mean cell area, median cell area, regression slope, absolute regression slope, and coefficient of determination (*R*^2^) were assessed using Pearson’s and Spearman’s correlation coefficients. Because of the asymmetric distribution of elemental concentrations, Pearson’s correlation analysis was also performed after logarithmic transformation of the concentration data. To account for multiple testing, (*p*)-values were adjusted using the Benjamini-Hochberg procedure.

In addition to the univariate correlation analyses, an exploratory multivariable linear regression model including the concentrations of Cr, V, W, Ti, Nb, and Mo as simultaneous predictors was fitted to assess their relationship with cell area. Given the limited number of samples (*n* = 22) relative to the number of predictors, the multivariable analysis was considered exploratory and was not used for confirmatory inference.

#### 2.3.3. Comparison of Alloying Groups

For group-based comparisons, the samples were classified according to the principal alloying element into Base, V, W, Mo, Nb, and Ti groups. Mixed-alloy samples E15, E16, and E22 were excluded from the primary group-based analysis. The Base group represented samples without intentionally increased contents of V, W, Mo, Nb, or Ti.

Differences among alloying groups were evaluated using one-way analysis of variance (ANOVA) and the non-parametric Kruskal–Wallis test. Where significant overall differences were detected, Tukey’s post hoc test was applied for pairwise group comparisons.

As a complementary analysis, samples were divided according to Ti content into a group with Ti ≥ 0.1% and a group comprising the remaining samples. Differences between these groups were evaluated using Welch’s test and the Mann–Whitney U test, and the magnitude of the group difference was quantified using Hedges’ g.

#### 2.3.4. Statistical Analysis of Alloying Effects

The experimental results were evaluated using descriptive statistics, statistical group comparisons, and multiple linear regression based on the general linear model (GLM) to investigate the relationships between alloying elements and the microstructural characteristics of the Fe–B–C alloy system. The selected experimental responses included the volume fraction of phases, the size of matrix cells, and the gradient of cell area variation across the casting cross-section.

The statistical analysis was carried out using the Statistica (Data Science Workbench, version 14; Cloud Software Group, Inc., Fort Lauderdale, FL, USA) software [40] and Microsoft Excel (Microsoft 365, Version 2610; Microsoft Corporation, Redmond, WA, USA) [41]. First, the main effects of the experimental factors were determined. The main effect represents the change in the response variable when a factor changes from its minimum to its maximum level while the other factors are kept at their average levels. This value therefore quantifies the direct influence of a particular alloying element on the investigated response.

To quantify the influence of the factors, a linear regression model was used to describe the relationship between the response and the experimental factors:

*Y = β*_0_* + β*_1_*X*_1_* + β*_2_*X*_2_* + β*_3_*X*_3_* + β*_4_*X*_4_* + … + β*_12_*X*_1_*X*_2_* + β*_13_*X*_1_*X*_3_* + β*_14_*X*_1_*X*_4_* + … + β*_56_*X*_5_*X*_6_* + ε* (2)

where:

- *Y* represents the investigated response (e.g., phase fraction, matrix cell area, or cell area gradient);
- *X*_1_, *X*_2_, *X*_3_, *X*_4_… represent the coded levels of the alloying elements (Cr = *X*_1_, V = *X*_2_, W = *X*_3_, Ti = *X*_4_, Nb = *X*_5_, Mo = *X*_6_);
- *β_i_* are the coefficients of the main effects;
- *β_j_* represent the coefficients of two-factor interactions;
- *ε* represents the random error of the model.

In addition to the main effects, interactions between factors were also evaluated. Interaction effects occur when the influence of one alloying element on the response depends on the concentration of another element. These interaction effects were identified through the regression model and evaluated using analysis of variance (ANOVA).

The statistical significance of individual effects was assessed based on *p*-values, where effects with *p* < 0.05 were considered statistically significant.

The outcome of this analysis allowed for the identification of the alloying elements and factor combinations that have the most significant influence on the formation of the Fe–B alloy microstructure, particularly with respect to phase fraction, matrix cell area, and the gradient of cell area variation across the casting cross-section.

## 3. Results

### 3.1. Results of Experimental Alloy Casting

The chemical composition of each sample, verified using the Bruker Q4 TASMAN optical emission spectrometer, is shown in Table 5.

A comparative assessment of the chemical composition in the matrix and boride phases was conducted for each sample using SEM-EDS analysis performed on a Tescan MIRA3 GMU scanning electron microscope. The results of the analysis are summarized in Table 6, where the weight percentages (wt.%) of the individual alloying elements are reported for samples E1–E22 in the respective phases. Phase identification is provided in the column “SL”, with the matrix region denoted as “M” and the boride-rich region denoted as “B”. For samples E4, E11, E12, E14, and E21, the presence of a carbide-rich phase, designated as “C”, was also identified by EDS analysis.

Carbon and boron were not evaluated among the alloying elements due to the low energy of their characteristic X-ray radiation, which is readily absorbed and therefore yields limited reliability in EDS measurements. Nickel was excluded because of peak overlap with neighbouring elements. For each sample, measurements were performed at least twice in each phase at different locations within the microstructure. The values reported in Table 6 represent the average of the obtained measurements.

### 3.2. Optical Microstructures of Experimental Alloys

**Figure 4:**
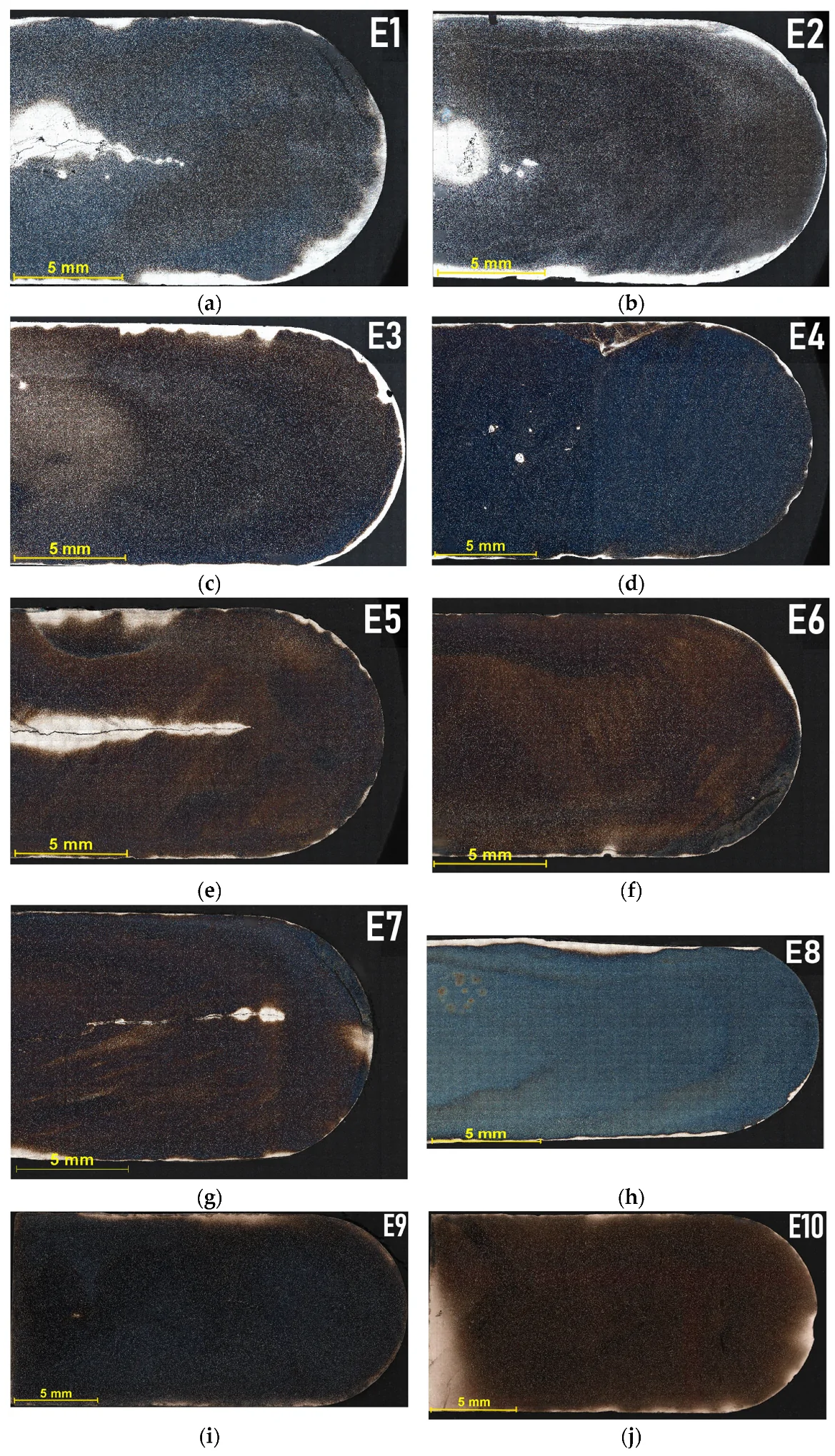

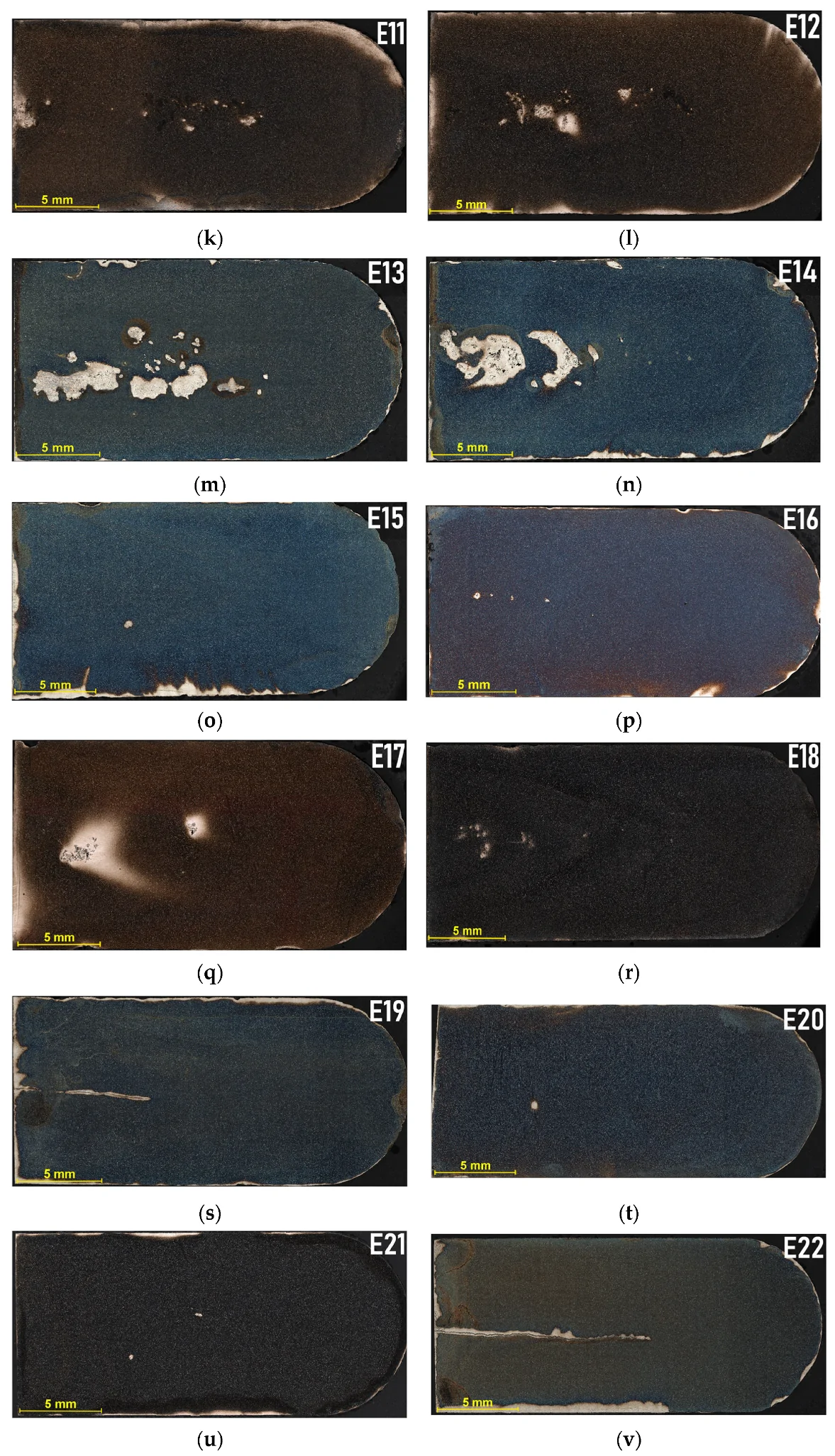
Full-cross-section microstructural mosaics of the analysed samples E1–E22 (**a**–**v**) etched with the etchant Klemm I.

The full-cross-section microstructural mosaics of samples E1–E22 are presented in Figure 4a–v. Variations in greyscale and colour intensity were observed among the samples, reflecting differences in etching response and local microstructural characteristics. Cracks were identified in samples E1, E5, E19, and E22, most likely as a result of casting and subsequent cooling. Bright regions were also observed in several samples, particularly E13 and E14, as well as near the sample–resin interface. These areas are presumed to be associated with the formation of oxide layers at the sample–resin boundary. These regions were associated with insufficiently revealed microstructure and resulted in localized data loss during automated image analysis. However, sufficient unaffected areas remained available for the overall microstructural evaluation.

### 3.3. Results of Greyscale-Based Image Classification

The analysed samples consisted of 638–1008 image frames, with the corresponding *.zvi file sizes ranging from approximately 19 to 31 GB. Samples containing fewer frames (638–750) generated datasets of approximately 19–22 GB, whereas samples containing 836–1008 frames resulted in larger datasets, typically ranging from 25 to 31 GB.

The mosaic dimensions varied among the analysed samples. Samples E1 and E2 consisted of 25 × 30 images (750 frames), E3 of 22 × 29 images (638 frames), and E4 of 24 × 32 images (768 frames). Samples E5–E7 had identical mosaic dimensions of 24 × 29 images (696 frames), whereas E8 consisted of 23 × 30 images (690 frames). Larger mosaics were acquired for E9 and E11 (23 × 39 images; 897 frames) and E10 (22 × 37 images; 814 frames). The largest mosaics were obtained for E12 and E14 (25 × 40 images; 1000 frames), while E13, E15, E17, and E18 contained between 936 and 966 frames. Samples E16 and E19 consisted of 22 × 38 images (836 frames), whereas E20–E22 contained 840–880 frames, depending on the mosaic dimensions.

The greyscale values measured across the entire sample image, including both the alloy and the surrounding mounting resin, showed considerable variability, ranging approximately from 3 to 167. In most samples, the maximum greyscale values were between 90 and 120, while E1, E2, and E5 exceeded 150. In contrast, greyscale values measured within the alloy itself were markedly less variable, with most samples exhibiting values between 30 and 60. Higher alloy greyscale values (approximately 60–90) were observed in E1–E3, E8, E15, and E16, whereas the lowest values (approximately 20–30) were recorded in E17–E21.

The number of segmentation procedures required varied among the samples. Three procedures were required for samples E1–E3, four procedures for E4 and E6–E8, and five procedures for E5. The remaining samples required either one (E9, E10, E12, E13, E15, E16 and E19–E21) or two (E11, E14, E17, E18 and E22) procedures.

### 3.4. Manual Verification of Segmentation Procedures

**Figure 5:**
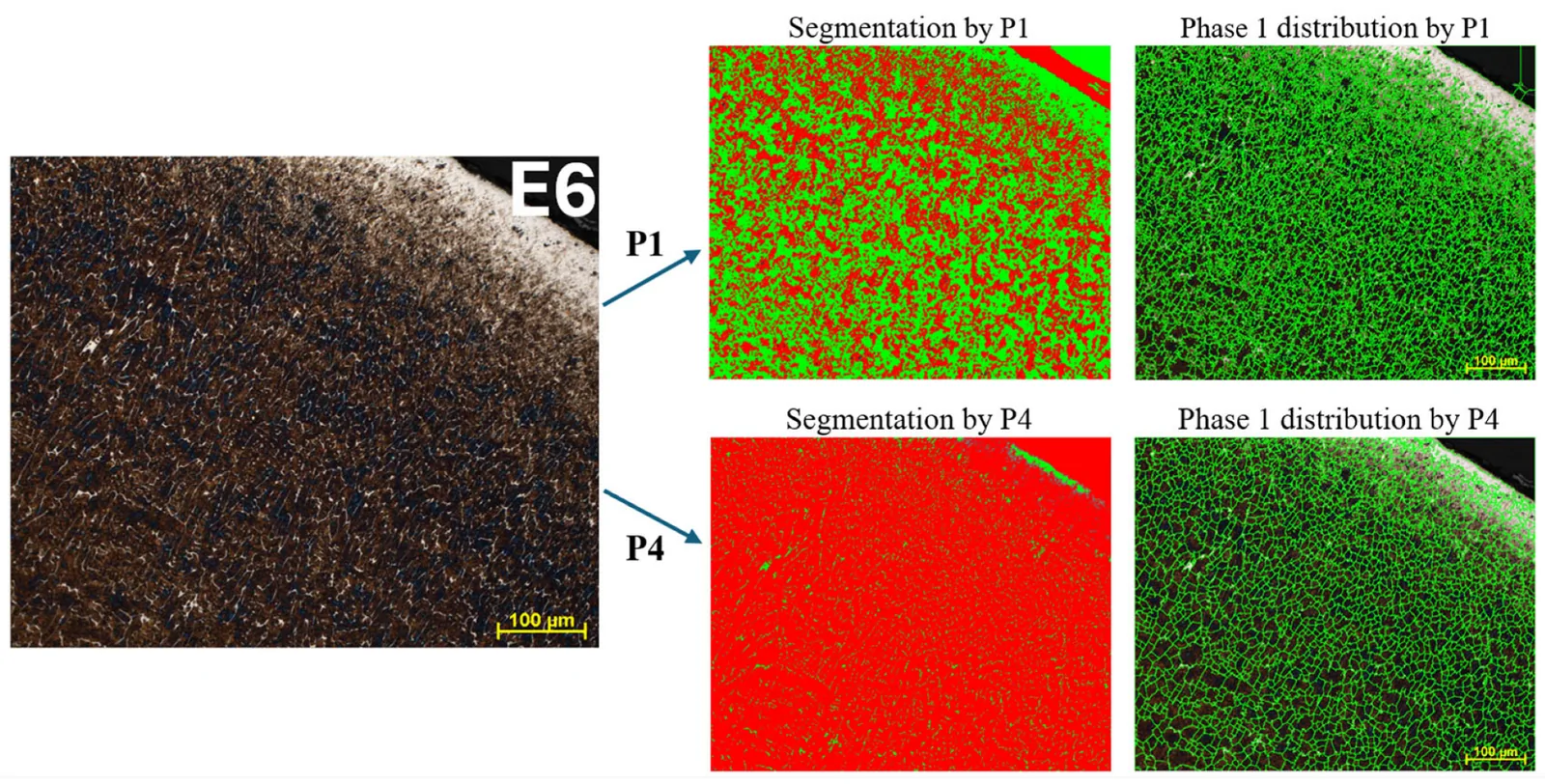
Comparison of segmentation procedures P1 and P4 applied to the same image of sample E6, demonstrating differences in phase separation accuracy.

The segmentation procedures defined based on greyscale intensity were subsequently verified manually to assess the accuracy of phase separation. Images corresponding to the limiting greyscale values of each procedure were inspected to confirm correct segmentation of Phase 1 (matrix) and Phase 2 (borides). The reliability of automated segmentation depended primarily on the contrast between the matrix and borides. High contrast enabled clear differentiation of both phases, whereas images with lower contrast required the removal of small incorrectly detected particles and image noise to achieve reliable phase separation. Figure 5 illustrates the effect of different segmentation settings using sample E6 as an example. Procedures P1 and P4 were applied to the same image, demonstrating that the selection of an appropriate procedure substantially affects phase separation. In this case, P1 provided more accurate segmentation of the matrix phase than P4.

**Figure 6:**
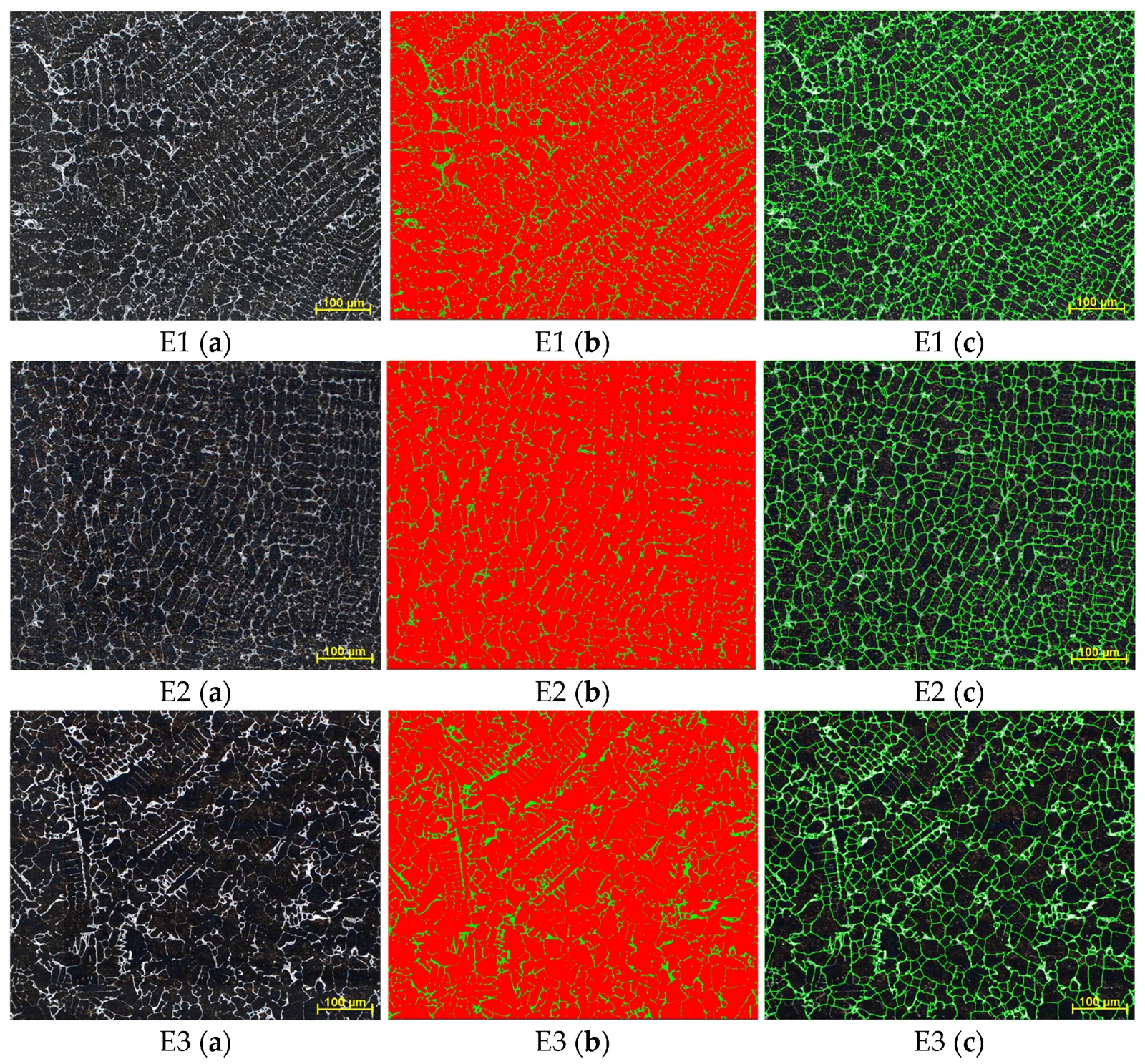

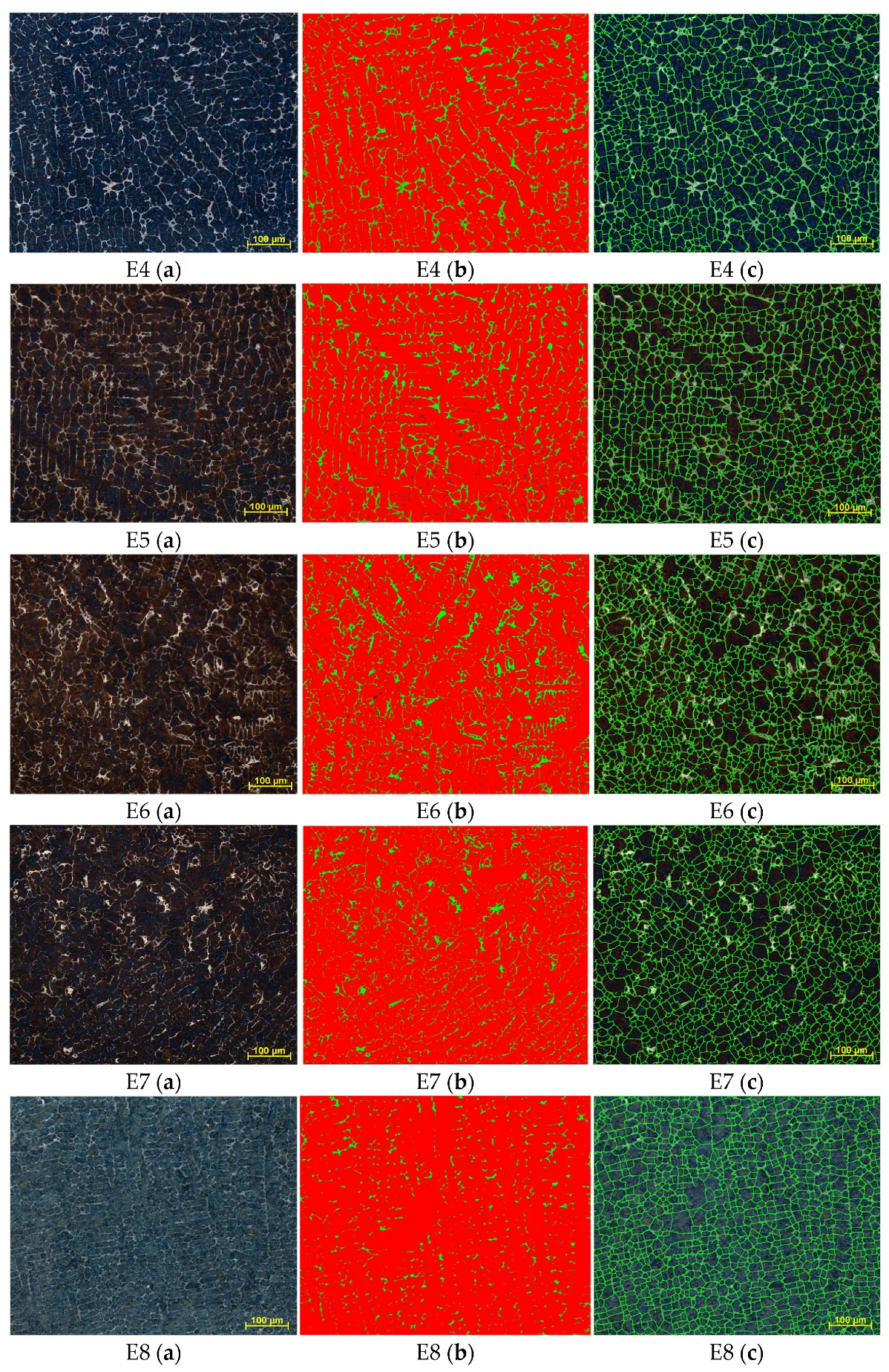

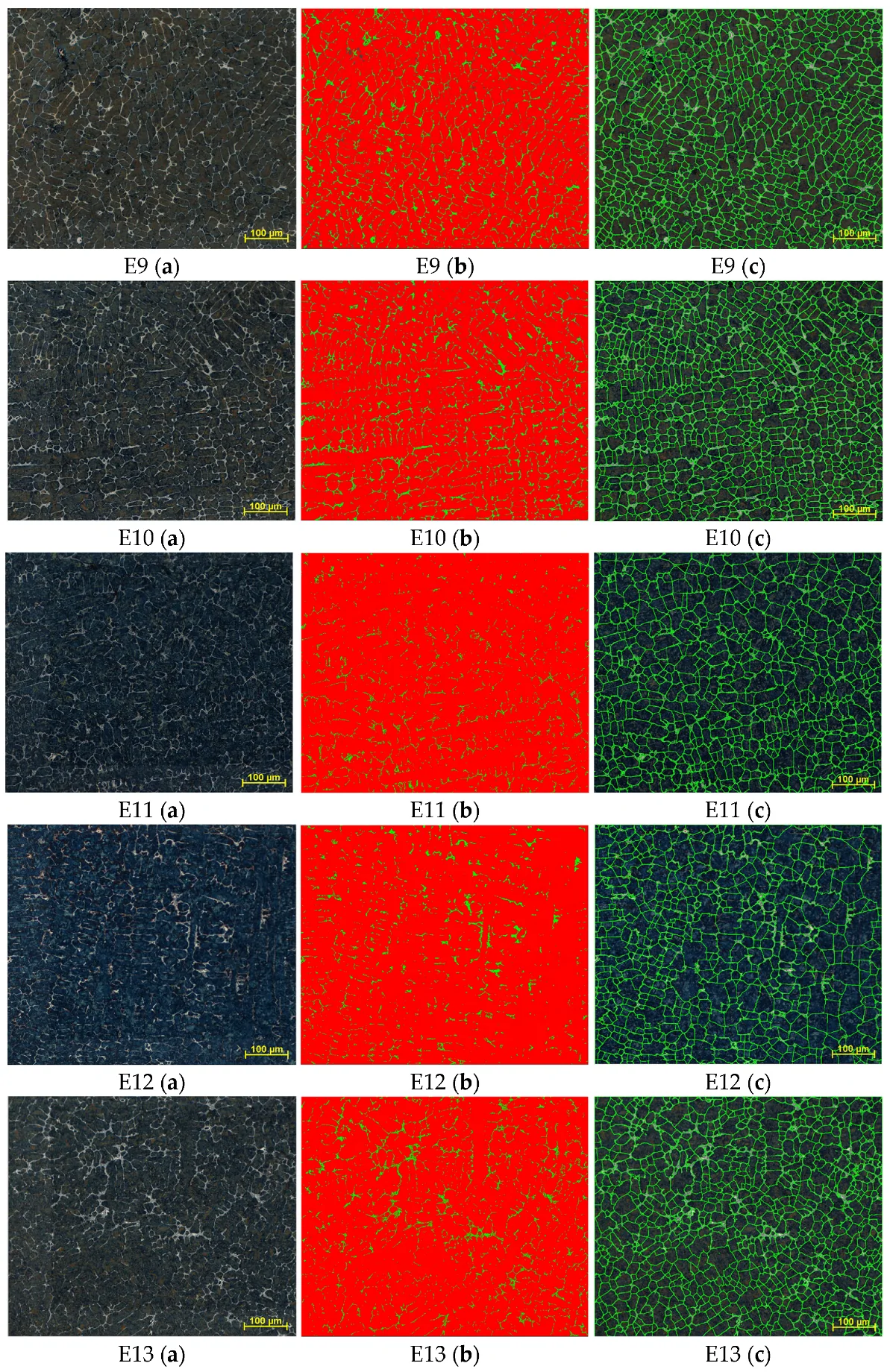

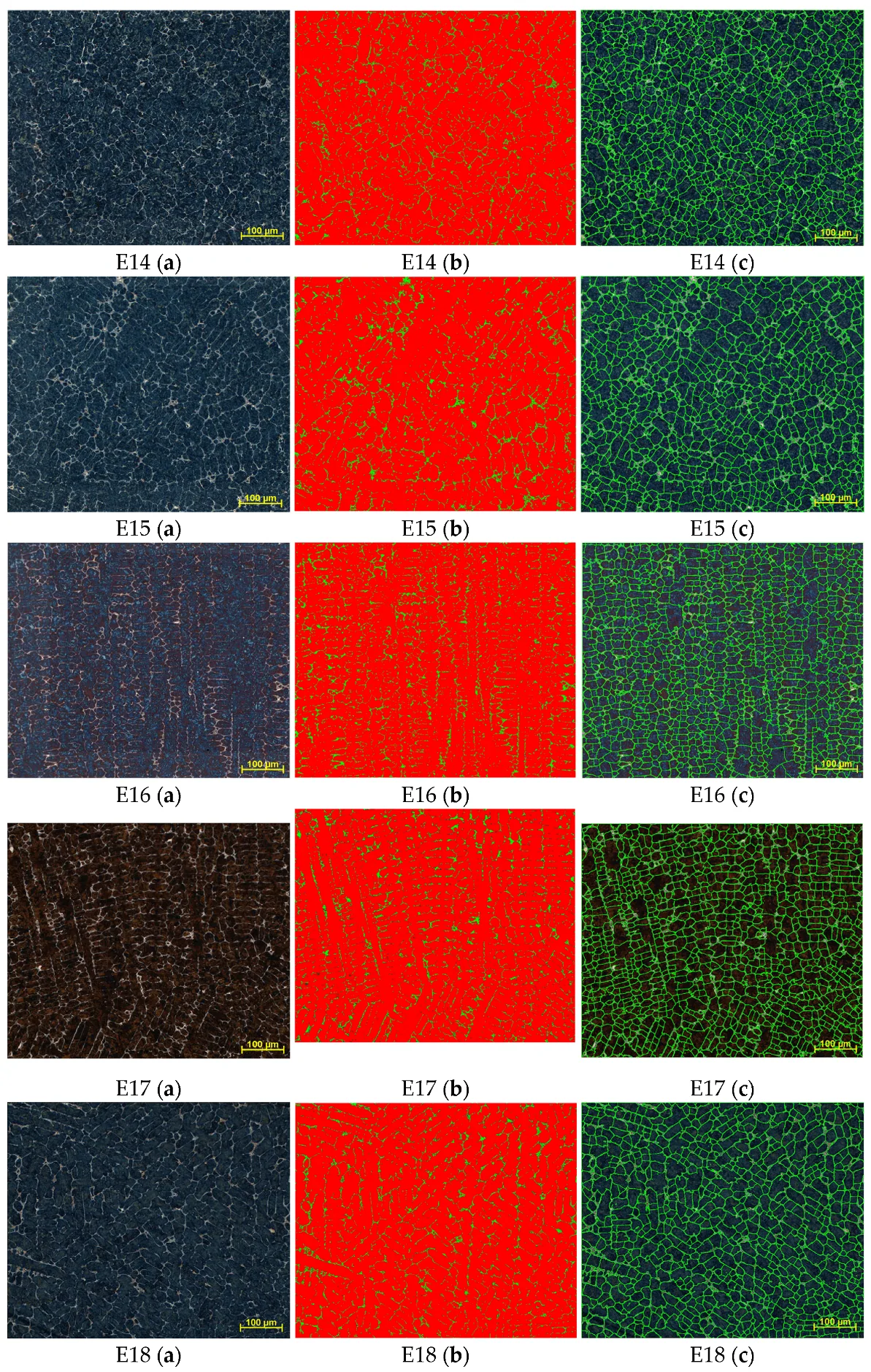

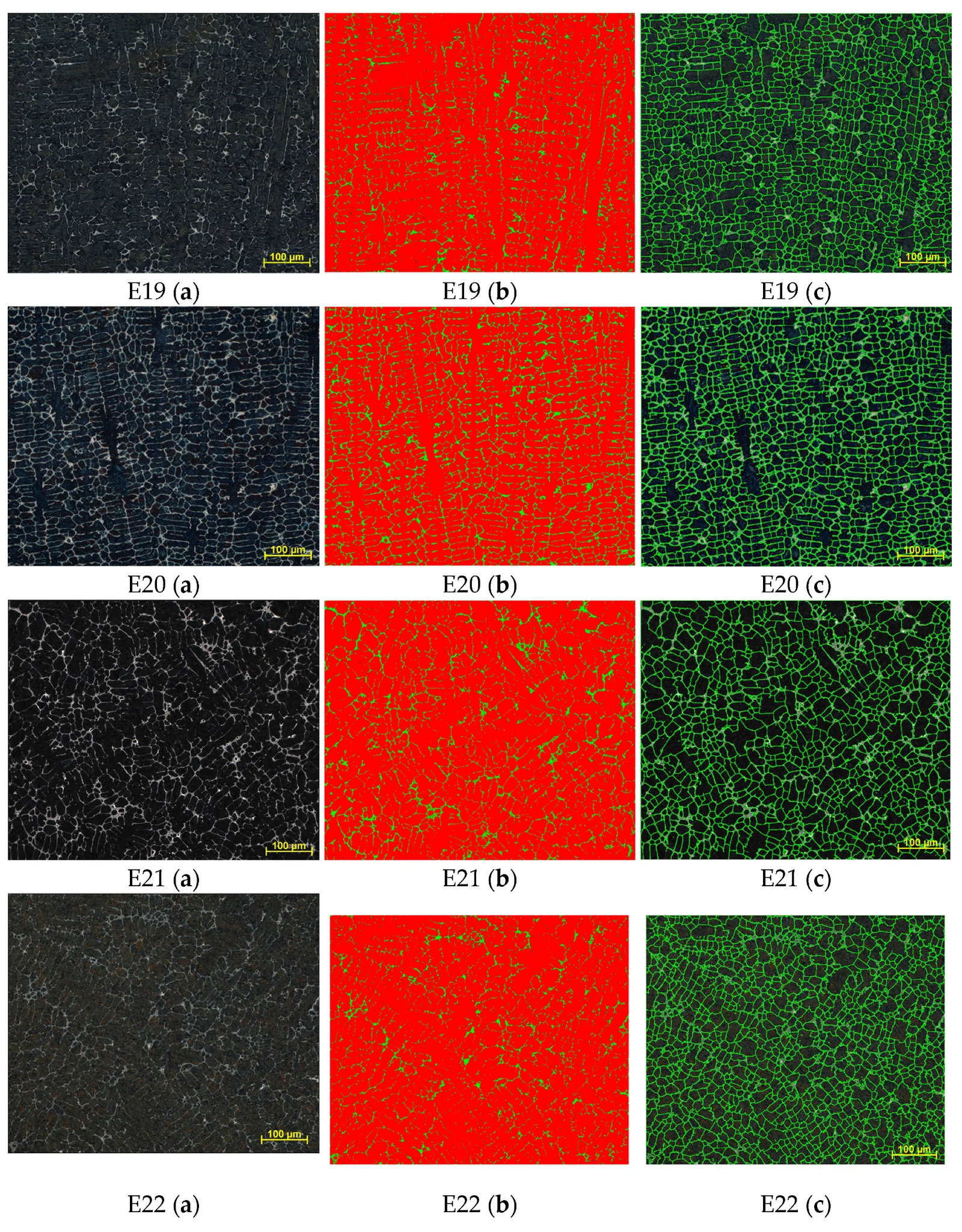
Microstructural analysis of samples E1–E22: (**a**) original microstructure after etching with Klemm I reagent, (**b**) segmentation into Phase 1—matrix (red) and Phase 2—borides (green), and (**c**) separation-line mask used for identification of individual matrix regions.

The microstructures of all analysed samples (E1–E22) etched with Klemm I reagent are presented in Figure 6. The optical micrographs were acquired at 200× magnification from the central region of each sample to ensure comparable observation conditions. For each sample, column (a) shows the original microstructure, column (b) shows the segmentation into Phase 1—matrix (red) and Phase 2—borides (green), and column (c) shows the separation-line mask used to identify individual matrix regions.

### 3.5. Cell Area Measurement and Data Processing

Manual measurements were used to determine the minimum and maximum cell area values (Amin and Amax) at individual depth levels (h0–h4), which were subsequently applied as filtering criteria for automated measurements. Comprehensive manual quantification, including measurements of at least 100 cells per image at each depth, was methodologically validated in our previous study [38] and was therefore not repeated in the present study.

Two automated cell area measurement steps were applied. In Automatic Measurement 1 (step 1), cell area data from individual images were filtered using the minimum and maximum values determined manually for the corresponding depth level. In Automatic Measurement 2 (step 2), global minimum and maximum limits derived from all evaluated depth levels (h0–h4) were applied, resulting in a unified dataset across the entire sample cross-section. A detailed description of the segmentation procedures and their verification through comparison with manual measurements is provided in Sections 2.3–2.6 of the previous study [38].

### 3.6. Cell Area Distribution for the Entire Sample

**Figure 7:**
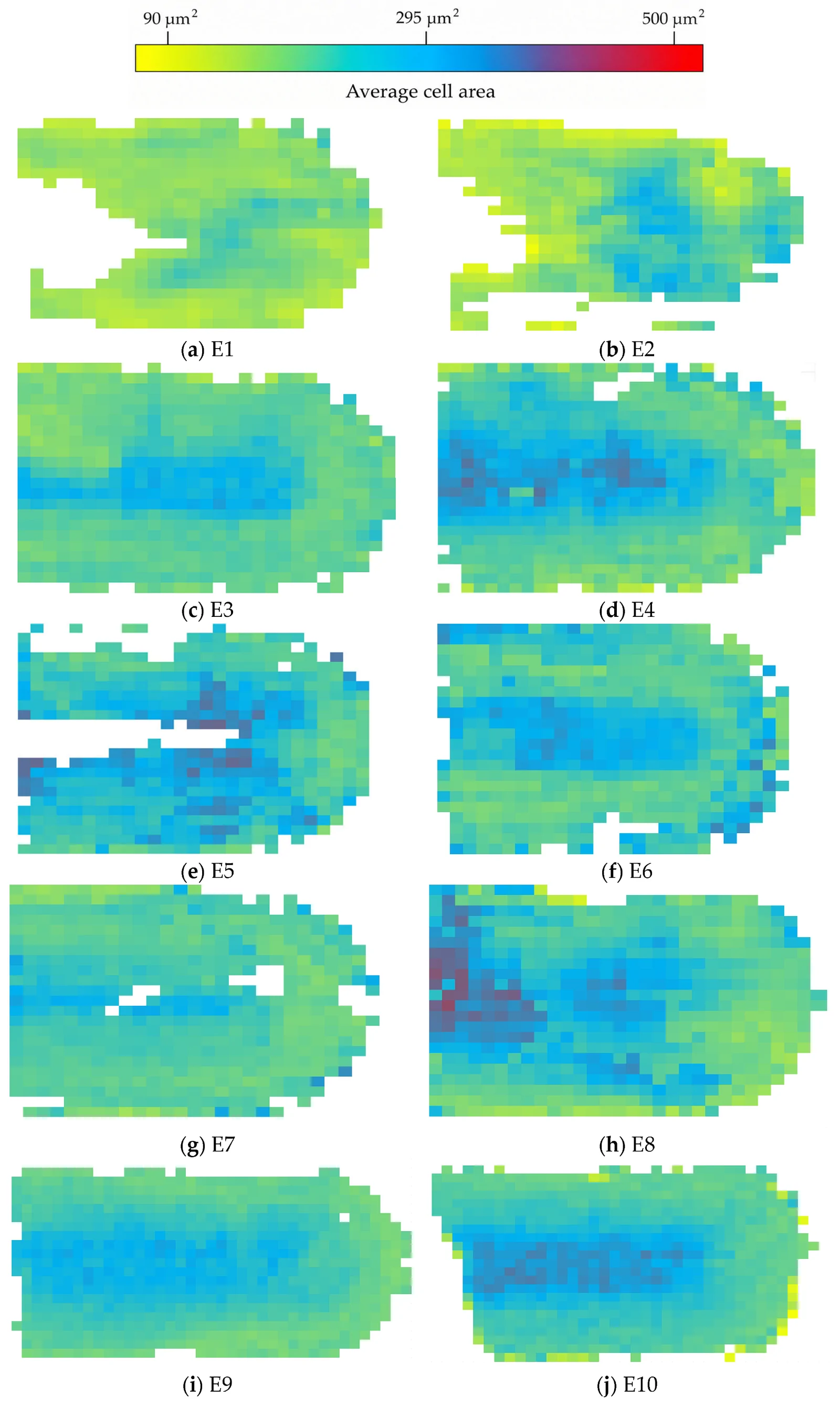

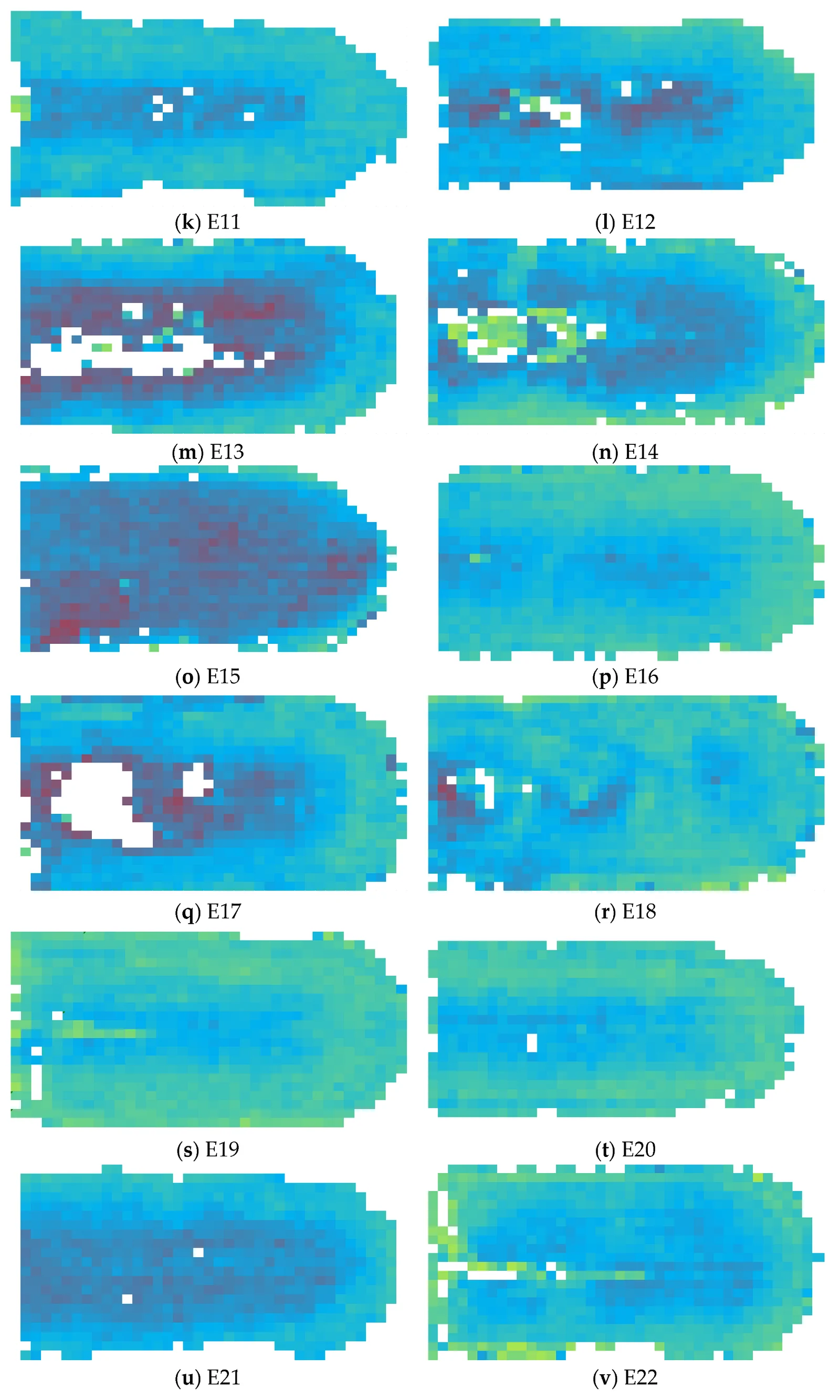
Resulting maps of average cell area within each image from E1 to E22.

Figure 7 presents the spatial distribution of cell area across the complete cross-section of all 22 experimental alloys. The maps reveal considerable differences both in the overall cell area level among the samples and in the spatial distribution within individual casting cross-sections. A general tendency towards larger cells in the interior regions and smaller cells towards the casting surface can also be observed.

**Figure 8:**
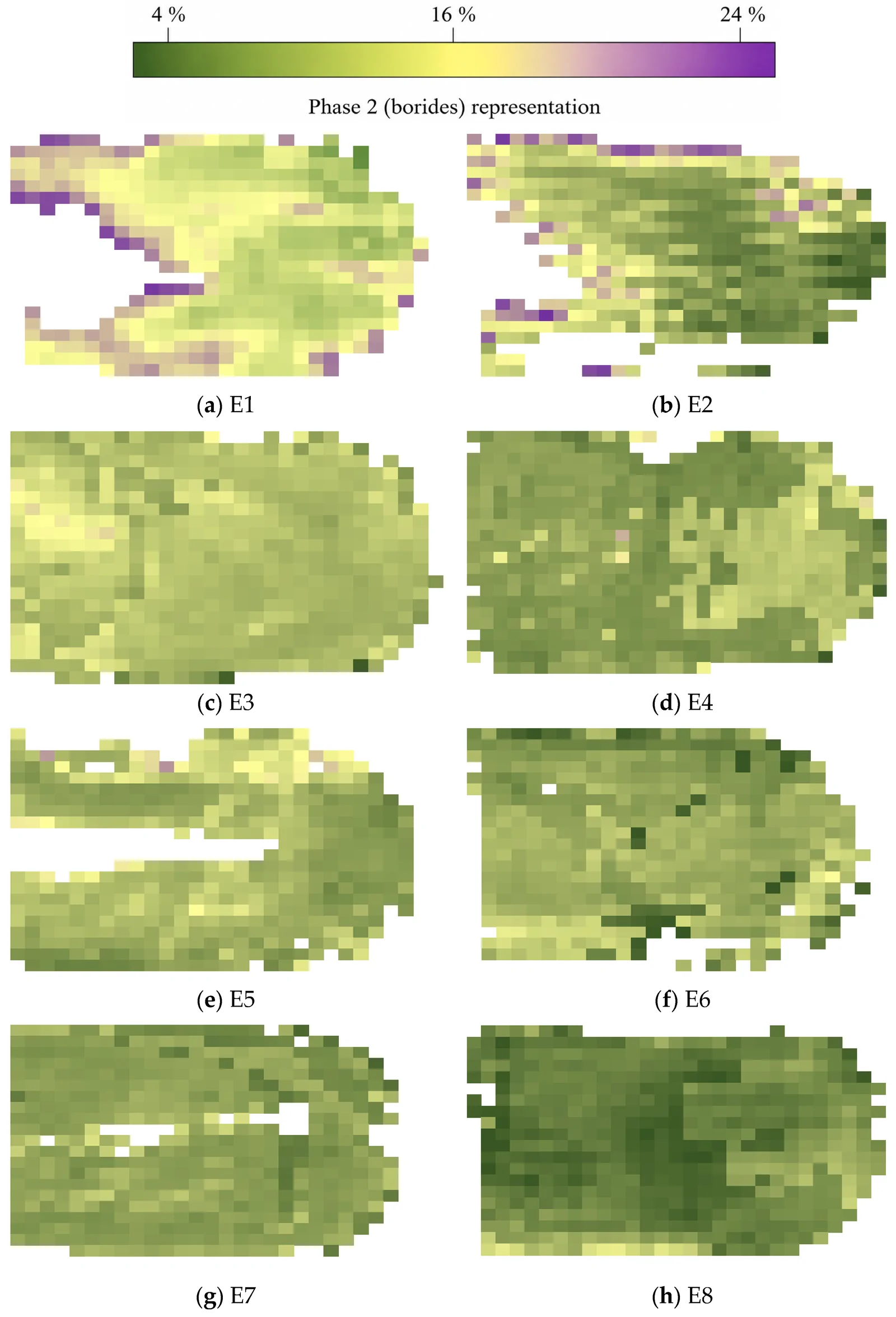

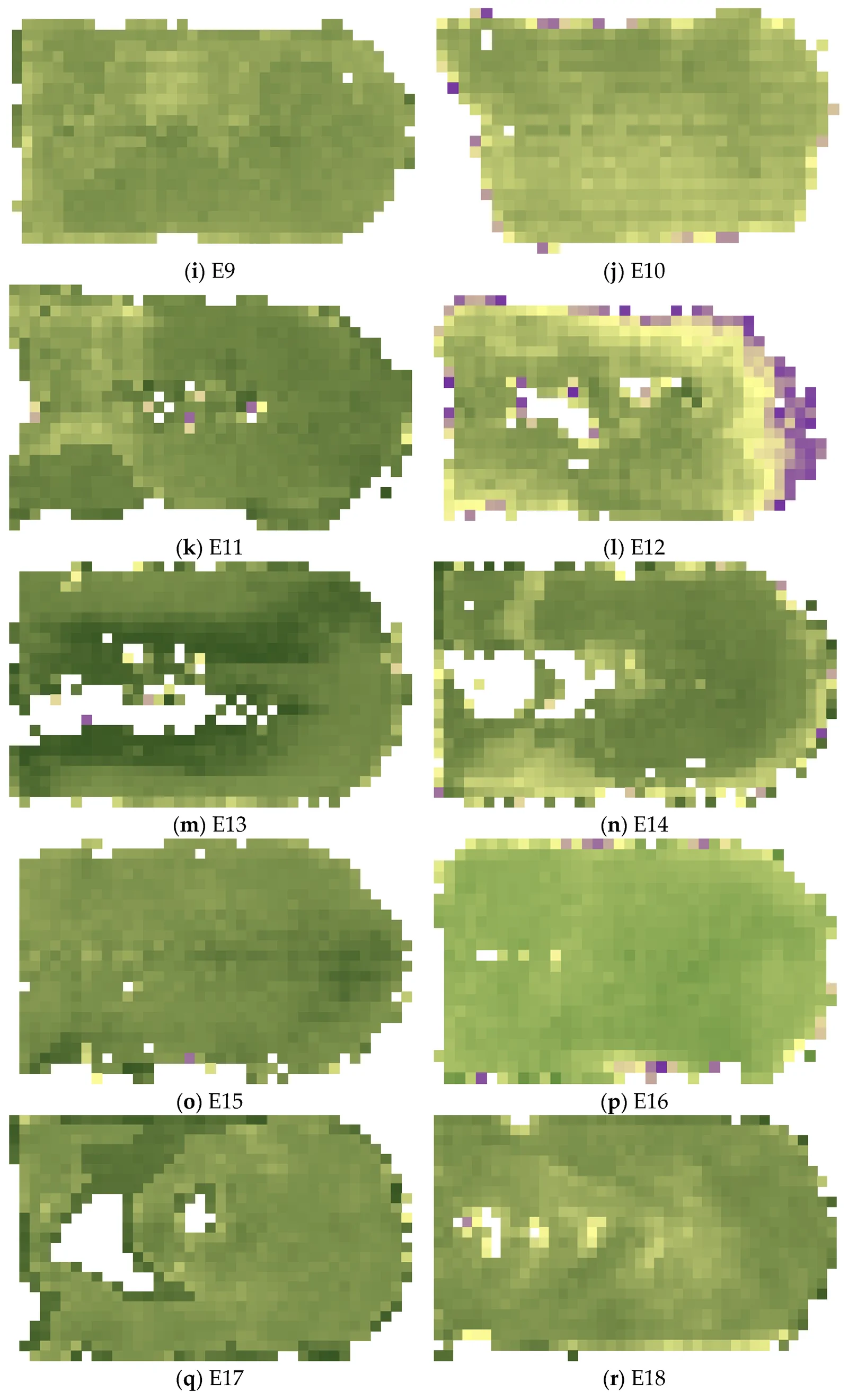

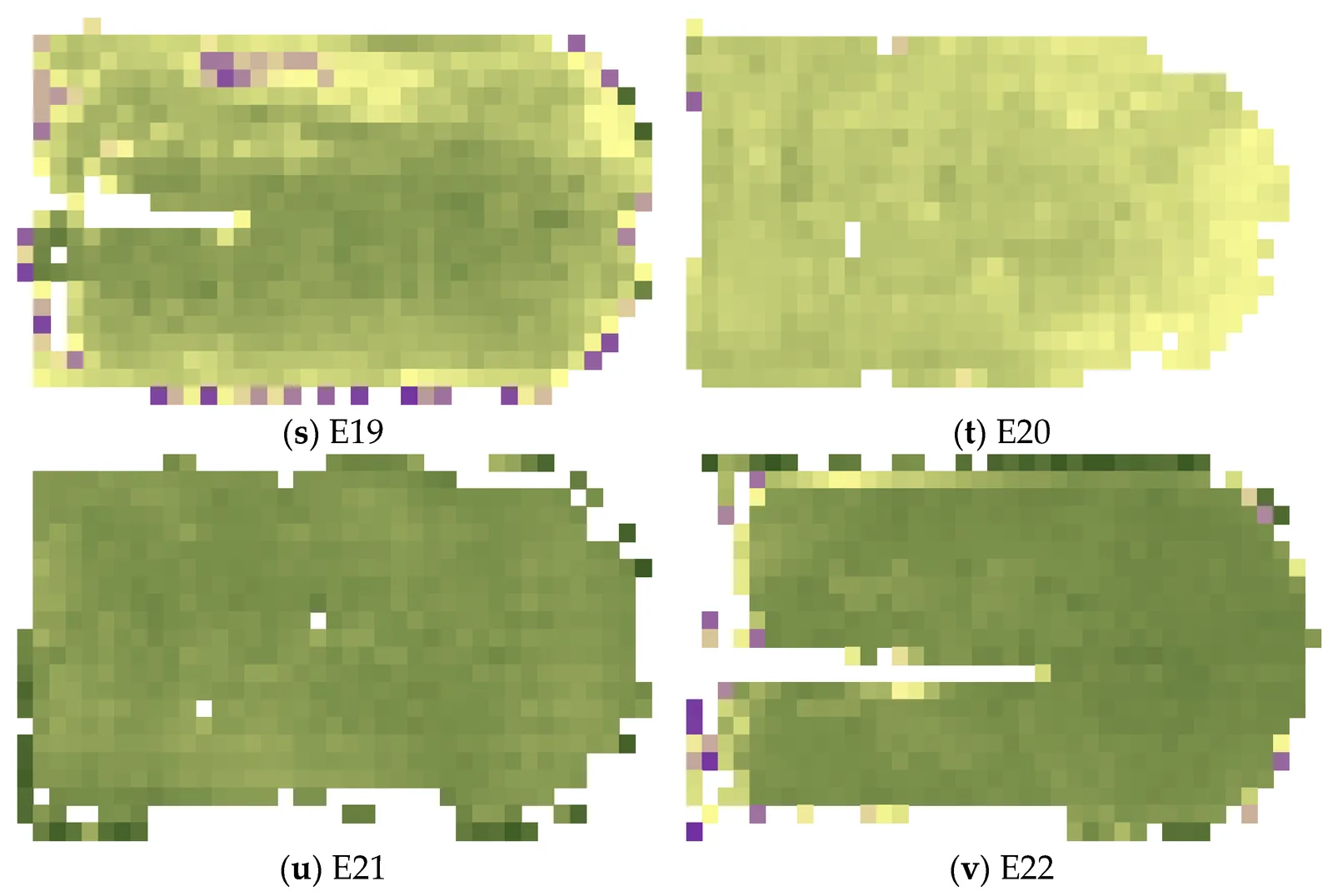
Resulting maps of Phase 2 (borides) representation within each image from E1 to E22.

In addition to cell area, the spatial distribution of Phase 2 (borides) was evaluated across the complete sample cross-sections (Figure 8). Each field represents the proportion of Phase 2 determined within the corresponding mosaic image. The maps therefore provide information on local variations in boride representation and enable comparison of its spatial distribution among the investigated alloys.

### 3.7. Statistical Evaluation of Cell Area Data

#### 3.7.1. Change in Cell Area from the Casting Centre to the Surface

For each predefined sample position, the arithmetic mean, median, standard deviation, and number of valid measurements were calculated. The dependence of cell area on distance from the core was subsequently evaluated by linear regression of the mean cell area at each position. Depending on the sample, approximately 10–13 positions were included in the regression (Table 7).

A negative regression slope was observed for all 22 samples, indicating that mean cell area decreased with increasing distance from the core. Statistical significance of the slope was assessed using a two-sided test of the null hypothesis that the slope was equal to zero.

In the primary analysis, the negative slope was statistically significant at the α = 0.05 level in 20 of the 22 samples (Table 7). The slopes for E3 (*p* = 0.064) and E14 (*p* = 0.059) were not statistically significant. For comparison, E22 yielded *p* = 0.035.

Regression based on position-wise means produced high coefficients of determination for several samples. For E1, based on 12 positions, *R*^2^ = 0.864 with *p* < 0.001; for E4, *R*^2^ = 0.894 with *p* < 0.001; and for E9, based on 11 positions, *R*^2^ = 0.953 with *p* < 0.001.

When all individual measurements were regressed against position, *R*^2^ values were lower than those obtained from regressions of position-wise means. Therefore, slopes derived from the mean values at individual positions were used primarily for subsequent comparisons among samples. The Theil-Sen slope was additionally calculated as a robust complementary estimator.

#### 3.7.2. Relationship Between Chemical Composition and Cell Area

The relationship between chemical composition and cell area parameters was evaluated for Cr, V, W, Ti, Nb, and Mo. In the primary analysis, values below the detection limit were replaced by one-half of the corresponding detection limit. Sensitivity analyses were additionally performed using replacement by zero and by the full detection limit.

The strongest correlations were observed for Ti. Spearman’s rank correlation coefficient between Ti content and mean cell area was *ρ* = 0.838. After correction for multiple testing using the Benjamini-Hochberg procedure, *q* < 0.001. For the relationship between Ti content and median cell area, *ρ* = 0.869 and *q* < 0.001 were obtained.

A significant association between Ti content and cell area was also obtained using Pearson correlation after logarithmic transformation of the concentrations. For mean cell area, *r* = 0.663 and *q* = 0.021 were obtained, whereas for median cell area, *r* = 0.734 and *q* = 0.0055 were obtained. All of these relationships were positive.

For Cr, V, W, Nb, and Mo, no statistically significant individual correlations with mean cell area, median cell area, regression slope, or the absolute slope value were observed after correction for multiple testing.

The concentrations of Cr, V, W, Ti, Nb, and Mo were simultaneously included in a multiple linear regression model (Table 8). The model for mean cell area yielded an adjusted coefficient of determination of *R*^2^ = 0.275. The standardized regression coefficient for Ti was *β* = 0.799 (*p* = 0.003), while for Nb, *β* = 0.519 (*p* = 0.032). Given the limited sample size (*n* = 22) relative to the six chemical predictors included simultaneously, the multivariable model was interpreted as exploratory and was not used for confirmatory inference.

#### 3.7.3. Comparison of Groups According to the Principal Alloying Element

For group-based comparisons, the samples were assigned to the Base, V, W, Mo, Nb, and Ti groups according to the principal alloying element. The Base group comprised E1 and E2, the V group E3 and E4, the W group E5-E8, the Mo group E9, E10, and E17-E20, the Nb group E11 and E12, and the Ti group E13, E14, and E21. Mixed-alloy samples E15, E16, and E22 were excluded from the group-based ANOVA (Table 9).

The mean cell areas were 198 µm^2^ for Base, 247 µm^2^ for V, 259 µm^2^ for W, 267 µm^2^ for Mo, 294 µm^2^ for Nb, and 311 µm^2^ for Ti (Table 9). One-way ANOVA revealed significant differences among groups for both mean cell area (*F* = 8.048, *p* = 0.001) and median cell area (F = 11.149, *p* < 0.001), while the corresponding Kruskal–Wallis tests yielded *p* = 0.027 for both variables (Table 10). No significant group differences were observed for the absolute mean regression slope or mean *R*^2^ (Table 10).

Tukey’s post hoc test identified significant differences in mean cell area for Base-W (difference = −61 µm^2^, *p* = 0.048), Base-Mo (−69 µm^2^, *p* = 0.014), Base-Nb (−96 µm^2^, *p* = 0.006), Base-Ti (−113 µm^2^, *p* <0.001), and V-Ti (−64 µm^2^, *p* = 0.047) (Table 11). For median cell area, significant differences were observed for Base-W (−59 µm^2^, *p* = 0.044), Base-Mo (−70 µm^2^, *p* = 0.009), Base-Nb (−97 µm^2^, *p* = 0.004), Base-Ti (−127 µm^2^, *p* <0.001), V-Ti (−81 µm^2^, *p* = 0.007), and W-Ti (−68 µm^2^, *p* = 0.007) (Table 11). Negative differences indicate that the first-listed group had a smaller cell area than the second-listed group; no significant difference was detected between Base and V.

In an additional comparison based on Ti content, samples with Ti ≥ 0.1% exhibited a mean cell area approximately 48 µm^2^ larger than the remaining samples. Welch’s test yielded *p* = 0.018, the Mann–Whitney U test *p* = 0.021, and Hedges’ g = 1.36. The median cell area was approximately 61 µm^2^ larger in the Ti ≥ 0.1% group, with *p* = 0.009 for Welch’s test, *p* = 0.021 for the Mann–Whitney U test, and Hedges’ *g* = 1.63.

### 3.8. Effect of Alloy Composition on Boride Morphology

**Figure 9:**
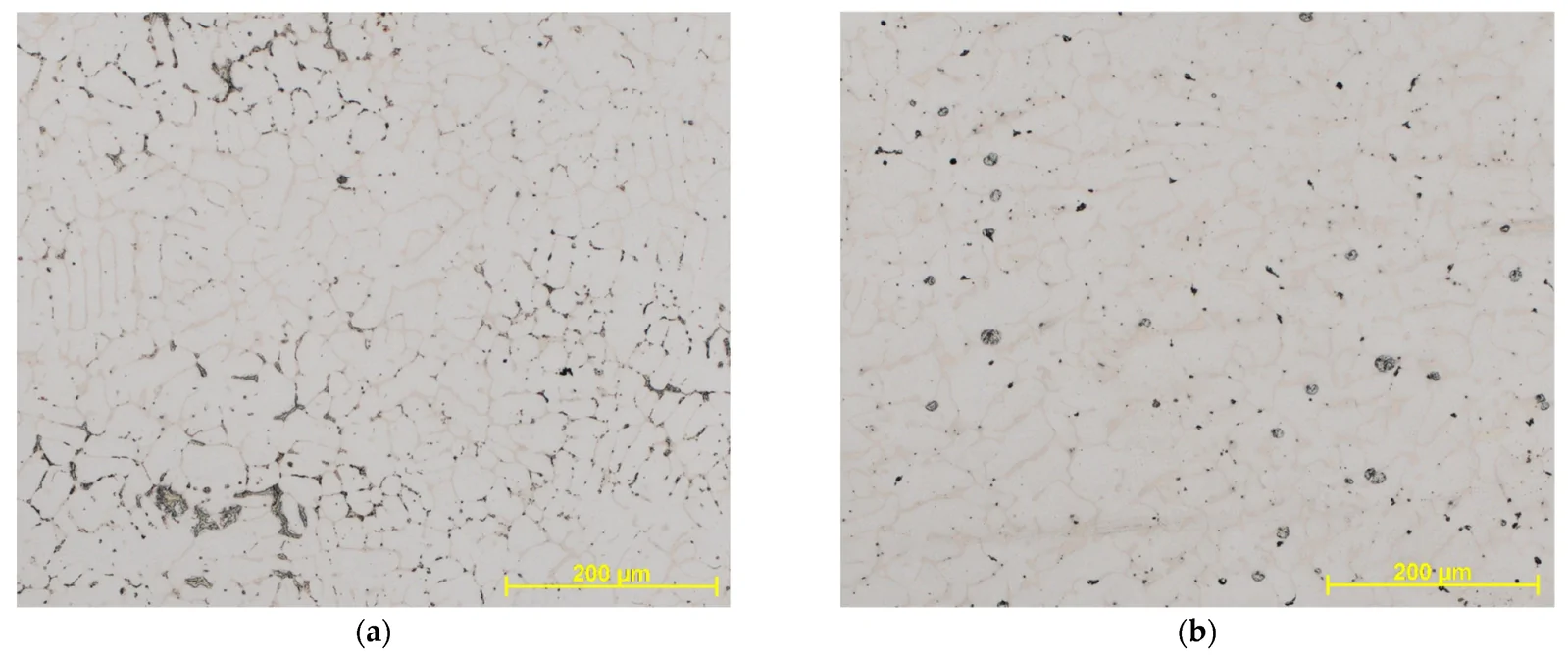
Representative optical micrographs of polished sample surfaces illustrating contrasting boride morphologies in the experimental alloys: (**a**) E13, with a predominantly network-like boride morphology, and (**b**) E16, with a strongly fragmented, fine network-like boride morphology.

In addition to the quantitative evaluation of cell area and the spatial distribution of the boride phase, the morphology of borides was examined in all 22 experimental alloys (E1–E22). Prior to optical microscopy, the sample surfaces were metallographically prepared, with final polishing performed using an OP-S colloidal silica suspension (Struers, Ballerup, Denmark). The assessment was based on optical micrographs acquired at 200× and 500× magnification and focused on the predominant boride morphology, the continuity of the boride network, and the presence of accompanying carbide-rich or other dark regions. The main morphological characteristics of the individual alloys are summarised in Table 12, providing a basis for evaluating the relationship between boride morphology and alloy composition. Representative examples of the contrasting boride morphologies are shown in Figure 9, illustrating the predominantly network-like structure of E13 and the strongly fragmented boride network of E16.

#### 3.8.1. Boride Morphology as a Function of Alloy Composition

The morphology of the boride phase varied considerably among the investigated alloys, ranging from interconnected network-like arrangements to polymorphic, rod-like, and highly fragmented morphologies. Since the B and C contents varied within relatively narrow ranges across the experimental series, the observed differences were evaluated primarily with respect to the major alloying additions and their interaction with Cr. Consistent with the cell area analysis, the alloys were classified into the Base, V, W, Mo, Nb, Ti, and combined-alloying groups.

##### Base Group

The base alloys were used to assess the effect of Cr in the absence of significant additions of the other investigated alloying elements. Both E1 and E2 exhibited predominantly network-like borides with moderate interconnectivity. Increasing the Cr content in E2 did not substantially alter the overall connectivity, although the borides became more polymorphic and locally branched compared with the more irregular network observed in E1. Thus, within the investigated compositional range, Cr alone produced only a limited change in the overall boride morphology.

##### Vanadium Group

A more pronounced morphological response to Cr was observed in the V-containing alloys. E3 (0.51 wt.% V, 0.05 wt.% Cr) exhibited predominantly fragmented and polymorphic borides with relatively low connectivity, whereas E4, with a comparable V content (0.52 wt.%) and higher Cr concentration (0.95 wt.%), showed a more developed network-like morphology with locally rod-like borides. The higher Cr content at approximately constant V concentration was therefore associated with increased boride connectivity. Compared with the Base group, this indicates that the effect of Cr on boride morphology depends on the accompanying alloying element.

##### Tungsten Group

The W-containing alloys were generally characterised by relatively fragmented boride morphologies. E5 and E7, containing lower Cr concentrations at comparable W levels, exhibited fragmented to fine network-like borides with low to moderate connectivity. E6 and E8, containing higher Cr concentrations, remained predominantly fragmented and polymorphic. Consequently, increasing the Cr content did not produce a systematic increase in boride connectivity in the W-containing alloys. The W group was therefore characterised mainly by disruption of the interconnected boride network, with no clear systematic effect of Cr within the investigated compositional range.

##### Mo Group

The Mo-containing alloys exhibited the widest range of boride morphologies. At approximately 0.8 wt.% Mo, E9 and E10 provide a direct comparison of the effect of Cr. E9 (0.45 wt.% Cr, 0.80 wt.% Mo) exhibited predominantly rod-like and fragmented borides with low connectivity, whereas E10 (1.00 wt.% Cr, 0.82 wt.% Mo) showed a substantially more developed network/intercrystalline morphology with medium to high connectivity. Thus, similarly to the V-containing alloys, higher Cr content at approximately constant Mo concentration was associated with increased boride connectivity.

The effect of Mo concentration at approximately constant Cr content was assessed using E17–E20. With increasing Mo content from 0.20 to 1.00 wt.%, the morphological evolution was non-monotonic. E17 and E18 exhibited predominantly polymorphic and fragmented borides, whereas E19 (0.60 wt.% Mo) showed locally more interconnected and elongated boride regions. At 1.00 wt.% Mo, E20 again exhibited a highly fragmented morphology with low connectivity, characterised by fine, discontinuous boride segments. These results indicate that the effect of Mo on boride morphology was not characterised by a simple progressive refinement or fragmentation within the investigated concentration range.

##### Nb Group

Both Nb-containing alloys retained a comparatively well-developed network component. E11 (1.03 wt.% Nb, 0.47 wt.% Cr) exhibited network-like borides with locally pronounced branched or fishbone-like morphology and medium to high connectivity. At a similar Nb concentration, increasing Cr to 1.02 wt.% in E12 resulted in a more polymorphic network with somewhat lower connectivity. In contrast to the V and Mo groups, the principal effect was therefore a change in local boride geometry and branching rather than a transition between strongly fragmented and interconnected morphologies.

##### Ti Group

The Ti-containing alloys showed a comparatively clear change in boride connectivity with composition. E13 (0.49 wt.% Cr, 0.28 wt.% Ti) exhibited a relatively regular network-like morphology with medium connectivity, forming a partially interconnected boride network, as illustrated in Figure 9a. E14, containing a similar Ti concentration (0.27 wt.%) but higher Cr content (0.98 wt.%), exhibited a finer and partially interrupted network. E21 (1.00 wt.% Cr, 0.20 wt.% Ti) showed predominantly fragmented borides with low connectivity and locally elongated features. Thus, unlike the V- and Mo-containing alloys, higher Cr contents in combination with Ti were associated with reduced continuity of the boride network.

##### Combined Alloying

Samples containing combined alloying elements provide further evidence that the morphology of borides cannot be predicted from the concentration of a single element. E15 and E16 contained comparable V and W concentrations, while the Cr content increased from 0.49 to 0.98 wt.%. Nevertheless, both alloys exhibited predominantly fine and strongly fragmented borides with low connectivity. The characteristic fragmented morphology of E16 is illustrated in Figure 9b. The investigated change in Cr concentration therefore did not substantially modify the morphology of the V–W-containing alloys.

E22 provides a useful comparison with E18. Both alloys contained approximately 0.5 wt.% Cr, 0.4 wt.% Mo, and 0.35 wt.% B, while E22 additionally contained 0.48 wt.% V. E18 exhibited predominantly polymorphic and fragmented borides with low to moderate connectivity, whereas E22 showed a more pronounced network-like component with medium connectivity and locally branched or fishbone-like features. Under these compositional conditions, the additional V content was therefore associated with greater development of the interconnected boride network.

To evaluate the relationship between boride-network characteristics and cell area, the specimens were first compared across four levels of visually assessed network connectivity: low (*n* = 7), low–medium (*n* = 5), medium (*n* = 8), and medium–high (*n* = 2). The corresponding group averages of the specimen mean cell areas were 263.6, 288.4, 262.8, and 271.0 µm^2^, respectively, while the group averages of the specimen median cell areas were 259.9, 283.6, 261.9, and 263.0 µm^2^. The Kruskal–Wallis test showed no significant differences among the four connectivity categories for either the specimen mean cell area (*H* = 2.158, *p* = 0.540) or the specimen median cell area (*H* = 1.404, *p* = 0.705). Furthermore, no significant monotonic association was found between increasing network connectivity and either the mean cell area (*ρ* = 0.088, *p* = 0.696) or median cell area (*ρ* = 0.084, *p* = 0.710).

As a complementary analysis of predominant morphology, the specimens were subsequently divided into network-dominant (*n* = 10) and fragmented/non-network-dominant (*n* = 12) groups. The network-dominant group exhibited an average specimen mean cell area of 264.4 µm^2^, compared with 273.9 µm^2^ for the fragmented/non-network-dominant group. The corresponding averages of the specimen median cell areas were 262.1 and 269.8 µm^2^, respectively. The Mann–Whitney U test showed no significant difference between the two morphological groups for either the specimen mean cell area (*U* = 57.0, *p* = 0.869) or the specimen median cell area (*U* = 59.0, *p* = 0.974). The corresponding effect sizes were negligible (Cliff’s *δ* = −0.050 and *δ* = −0.017, respectively). Within the limits of the present semiquantitative classification, neither visually assessed boride-network continuity nor predominant morphology showed a statistically supported relationship with cell area.

## 4. Discussion

In our study, full-area optical microscopy was combined with quantitative cell area analysis and qualitative assessment of boride morphology in 22 experimental Fe–B–C alloys. The spatially resolved evaluation provided information on microstructural variations across the casting cross-section. It also enabled comparison of matrix cell area and boride morphology among alloys with different chemical compositions.

Statistical analysis showed negative regression slopes for all 22 samples, indicating a decrease in cell area from the core towards the casting surface. This trend was statistically significant in 20 samples, with only E3 and E14 showing non-significant slopes. Regressions based on position-wise mean cell areas generally provided higher coefficients of determination than those based on individual measurements.

The strongest relationship between chemical composition and cell area was observed for Ti, which showed significant positive correlations with both mean and median cell area. No significant individual correlations were found for the other alloying elements after correction for multiple testing. The Ti group also exhibited the largest cell areas, while the multivariable model identified positive associations for both Ti and Nb. Despite significant differences in mean and median cell area among the alloying groups, no significant differences were found in the magnitude of the radial gradient. These findings suggest that alloy composition was associated with overall cell area, while a similar spatial trend was observed across the investigated alloys. The observed positive association between Ti content and cell area highlights the influence of alloy composition on the spatial arrangement of the boride network. Unlike conventional crystallographic grain area, the evaluated cell area characterises regions enclosed by boride phases and therefore reflects a different aspect of microstructural evolution. Previous studies have demonstrated that Ti can modify boride formation and morphology in Fe–B alloys. Ren et al. (2021) [42] reported refinement and fragmentation of the borocarbide network associated with TiC formation, while Skałoń et al. (2019) [43] described TiB_2_ formation and changes in Fe_2_B distribution in Fe–B alloys. These findings provide a possible explanation for the observed relationship between Ti content and cell area, suggesting that Ti-related changes in boride formation and spatial distribution may contribute to the measured differences. However, the observed statistical correlation alone does not establish a direct causal relationship.

In addition to the differences in matrix cell area, the investigated alloys exhibited considerable variation in boride morphology. Zhang et al. (2020) [18] reported that Fe–Si–Mn–Cr–B alloys containing 0.5 wt.% B exhibited discontinuous rod-like and polymorphic borides embedded within a continuous metallic matrix. At higher B contents (1.0–2.0 wt.%), network-like and fishbone-like morphologies became more pronounced. In the present study, network-like borides were also observed at lower B contents of approximately 0.35 wt.%, alongside rod-like, polymorphic, and strongly fragmented morphologies. However, direct comparison with Zhang et al. [18] is limited by differences in the investigated alloy systems. As the B and C contents varied only within narrow ranges, the observed morphological differences were further examined in relation to Cr and the accompanying alloying additions.

Previous studies have reported that Cr can modify eutectic boride morphology towards a more discontinuous network [22,23,26], while combined Cr–V and W additions have been associated with suppression of continuous or preferentially oriented Fe_2_B growth [8,9,25]. The present results showed different morphological responses to Cr depending on the accompanying alloying elements. The base alloys E1–E2 showed only limited sensitivity to increasing Cr. In contrast, higher Cr content was associated with greater boride connectivity in the V-containing pair E3–E4 and the Mo-containing pair E9–E10. The opposite tendency was observed in the Ti-containing pair E13–E14, where higher Cr content was associated with a finer and more discontinuous boride network. E21 also exhibited strongly fragmented borides at the higher Cr level. The W-containing alloys E5–E8 were predominantly fragmented, although their local morphological variability prevented identification of a simple Cr-dependent trend. In the V–W alloys E15–E16, the strongly fragmented morphology persisted at both investigated Cr concentrations.

Further morphological differences were observed in the Mo concentration series E17–E20. At nearly constant Cr content, the boride network became locally more interconnected at intermediate Mo concentrations. At the highest Mo content, E20 exhibited a strongly fragmented morphology with low connectivity. The morphological response to increasing Mo content was therefore non-monotonic within the investigated concentration range.

Overall, these observations suggest that boride morphology cannot be explained by Cr concentration alone. The combination and concentration of the accompanying alloying elements must also be considered. The qualitative assessment of boride morphology thus complements the quantitative evaluation of matrix cell area and its spatial distribution, allowing both characteristics to be compared among alloys with different chemical compositions.

Furthermore, the statistical analysis did not reveal a significant relationship between the visually assessed boride-network connectivity and the measured cell area. This finding indicates that cell area and boride morphology represent complementary microstructural characteristics and should be evaluated separately.

## 5. Conclusions

This study investigated the effects of Cr, V, W, Ti, Nb, and Mo additions on matrix cell area, its spatial distribution, and boride morphology in 22 experimental Fe–B–C cast alloys containing approximately 0.35 wt.% B. Full-area optical microscopy combined with automated image analysis enabled the evaluation of microstructural variations across the casting cross-sections and the comparison of alloys with different chemical compositions. The main findings of this study are as follows:

- Full-area optical microscopy revealed a decrease in matrix cell area from the casting core towards the surface in all 22 alloys, with statistically significant gradients in 20 samples.
- Mean and median cell areas differed significantly among the alloying groups, whereas no significant differences were detected in the magnitude of the radial gradient.
- Ti showed the strongest positive association with matrix cell area. The exploratory multivariable analysis also identified a positive association for Nb.
- Network-like, rod-like, polymorphic, and strongly fragmented boride morphologies were observed at B contents of approximately 0.35 wt.%.
- The morphological response to increasing Cr content differed among the alloying groups, while the Mo concentration series exhibited a non-monotonic response in boride-network connectivity.

The results demonstrate that full-area optical microscopy, combined with quantitative cell area analysis and qualitative boride morphology assessment, provides a useful approach for comparing microstructural characteristics and their spatial distribution in Fe–B–C cast alloys.

## Figures and Tables

**Table 1:** Chemical compositions (wt.%) of the ferroalloys determined by EDS and of C35 steel determined by OES [38].

Ferroalloys	B	C	Cr	V	W	Ti	Nb	Mo	Mn	Si	Ni	Cu	Al	Fe
FeB	18.49	0.31	-	-	-	-	-	-		0.43	-	-	-	Bal.
FeCr LC	-	0.06	69.49	-	-	-	-	-	-	1.32	-	-	-	Bal.
FeW	-	0.36	-	-	76.64	-	-	-	-	0.35	-	-	-	Bal.
FeV	-	0.25	-	80.73	-	-	-	-	1.13	1.25	-	-	-	Bal.
FeTi	-	0.13	-	2.96	-	69.51	-	-	-	0.21	-	-	-	Bal.
FeNb	-	0.13	-	-	-	-	65.33	-	-	2.36	-	-	-	Bal.
FeMo	-	0.10	-	-	-	-	-	64.39	-	1.49	-	-	-	Bal.
Steel C35	-	0.37	0.05	-	-	-	-	-	0.70	0.21	0.06	1.18	0.03	Bal.

**Table 2:** Composition ranges and investigated concentration levels of the alloying elements used in the DoE design.

Element	Low (wt.%)	High (wt.%)	Investigated Levels (wt.%)
Cr	0.4	1	0.40, 1.00
V	0.2	1	0.20, 0.50, 0.80, 1.00
W	0.2	1	0.20, 0.40, 0.50, 0.60, 0.80, 1.00
Mo	0.2	1.2	0.20, 0.40, 0.60, 0.80, 1.00, 1.20
Nb	0.2	1.2	0.20, 0.40, 0.60, 0.80, 1.00, 1.20
Ti	0.1	0.3	0.10, 0.30

**Table 3:** Phase compositions of the 22 experimental alloys obtained by JMatPro software.

Sample	Target Alloy Chemical Composition	Martensite Phase 1	Borides	Carbides	SUM Phase 2
E1	Fe_B04_Cr04_C035	91.7	8.3	0.0	8.3
E2	Fe_B04_Cr1_C035	90.6	6.8	2.6	9.4
E3	Fe_B04_V05_C035	91.8	7.5	0.7	8.2
E4	Fe_B04_Cr1_V05_C035	90.2	9.1	0.7	9.8
E5	Fe_B04_Cr04_W05_C035	89.8	4.2	6.0	10.2
E6	Fe_B04_Cr1_W05_C035	88.8	3.8	7.5	11.3
E7	Fe_B04_Cr04_W05_C035	90.0	3.3	6.7	10.0
E8	Fe_B04_Cr1_W05_C035	89.5	4.5	6.0	10.5
E9	Fe_B04_Cr04_Mo08_C035	92.1	7.9	0.0	7.9
E10	Fe_B04_Cr1_Mo08_C035	91.2	8.8	0.0	8.8
E11	Fe_B04_Cr04_Nb1_C035	90.4	8.5	1.1	9.6
E12	Fe_B04_Cr1_Nb1_C035	89.7	9.3	1.0	10.3
E13	Fe_B04_Cr04_Ti03_C035	91.1	8.3	0.6	8.9
E14	Fe_B04_Cr1_Ti03_C035	91.2	8.2	0.6	8.8
E15	Fe_B04_Cr04_V05_W05_C035	91.2	8.1	0.7	8.8
E16	Fe_B04_Cr1_V05_W05_C035	91.0	8.2	0.8	9.0
E17	Fe_B04_Cr04_Mo02_C035	91.2	6.9	1.9	8.8
E18	Fe_B04_Cr04_Mo04_C035	92.0	7.7	0.3	8.0
E19	Fe_B04_Cr04_Mo06_C035	91.6	8.4	0.0	8.4
E20	Fe_B04_Cr04_Mo1_C035	92.0	8.0	0.0	8.0
E21	Fe_B04_Cr1_Ti02_C035	90.2	7.1	2.8	9.8
E22	Fe_B04_Cr04_V05_Mo05_C035	92.0	7.3	0.7	8.0

**Table 4:** Target chemical compositions of the experimental alloys (E1–E22) and the corresponding calculated amounts of ferroalloys and C35 steel (in grams) used for the preparation of the casting charges.

Sample	Target Alloy Chemical Composition	FeB	FeCr	FeV	FeW	FeMo	FeNb	FeTi	C35
E1	Fe_B04_Cr04_C035	11 g	3 g	-	-	-	-	-	492 g
E2	Fe_B04_Cr1_C035	11 g	7 g	-	-	-	-	-	483 g
E3	Fe_B04_V05_C035	11 g	-	3 g	-	-	-	-	487 g
E4	Fe_B04_Cr1_V05_C035	11 g	7 g	3 g	-	-	-	-	488 g
E5	Fe_B04_Cr04_W05_C035	11 g	3 g	-	3 g	-	-	-	492 g
E6	Fe_B04_Cr1_W05_C035	11 g	7 g	-	3 g	-	-	-	491 g
E7	Fe_B04_Cr04_W05_C035	11 g	3 g	-	3 g	-	-	-	492 g
E8	Fe_B04_Cr1_W05_C035	11 g	7 g	-	3 g	-	-	-	491 g
E9	Fe_B04_Cr04_Mo08_C035	11 g	3 g	-	-	6 g	-	-	491 g
E10	Fe_B04_Cr1_Mo08_C035	11 g	7 g	-	-	6 g	-	-	480 g
E11	Fe_B04_Cr04_Nb1_C035	11 g	3 g	-	-	-	7.5 g	-	483 g
E12	Fe_B04_Cr1_Nb1_C035	11 g	7 g	-	-	-	7.5 g	-	489 g
E13	Fe_B04_Cr04_Ti03_C035	11 g	3 g	-	-	-	-	2 g	489 g
E14	Fe_B04_Cr1_Ti03_C035	11 g	7 g	-	-	-	-	2 g	490 g
E15	Fe_B04_Cr04_V05_W05_C035	11 g	3 g	3 g	3 g	-	-	-	488 g
E16	Fe_B04_Cr1_V05_W05_C035	11 g	7 g	3 g	3 g	-	-	-	493 g
E17	Fe_B04_Cr04_Mo02_C035	11 g	3.3 g	-	-	1.5 g	-	-	492 g
E18	Fe_B04_Cr04_Mo04_C035	11 g	3.5 g	-	-	4.4 g	-	-	499 g
E19	Fe_B04_Cr04_Mo06_C035	11 g	3.5 g	-	-	5 g	-	-	491 g
E20	Fe_B04_Cr04_Mo1_C035	11 g	3 g	-	-	7.5 g	-	-	481 g
E21	Fe_B04_Cr1_Ti02_C035	11 g	7 g	-	-	-	-	1.6 g	493 g
E22	Fe_B04_Cr04_V05_Mo05_C035	11 g	3 g	3 g	-	3 g	-	-	492 g

**Table 5:** The chemical compositions (wt. %) of cast steels for creation of the samples E1 to E22.

Sample	B	C	Cr	V	W	Ti	Nb	Mo	Mn	Si	Ni	Fe
E1	0.35	0.35	0.52	<0.005	<0.010	0.002	0.0003	<0.010	0.650	0.260	0.061	Bal.
E2	0.36	0.36	1.07	<0.005	<0.010	0.002	<0.010	<0.010	0.619	0.275	0.063	Bal.
E3	0.35	0.33	0.05	0.51	<0.010	0.002	0.003	<0.010	0.613	0.253	0.055	Bal.
E4	0.38	0.33	0.95	0.52	<0.010	0.003	0.005	0.011	0.647	0.229	0.062	Bal.
E5	0.36	0.35	0.47	<0.005	0.507	0.002	<0.010	0.020	0.652	0.234	0.058	Bal.
E6	0.38	0.37	1.02	<0.005	0.490	0.002	<0.010	0.014	0.652	0.258	0.060	Bal.
E7	0.34	0.35	0.59	<0.005	0.529	0.002	<0.010	0.013	0.652	0.242	0.059	Bal.
E8	0.37	0.35	1.07	<0.005	0.433	0.003	<0.010	0.012	0.648	0.245	0.061	Bal.
E9	0.36	0.33	0.45	<0.005	<0.010	0.002	<0.010	0.798	0.658	0.248	0.059	Bal.
E10	0.40	0.33	1.00	<0.005	<0.010	0.003	<0.010	0.823	0.661	0.248	0.060	Bal.
E11	0.36	0.35	0.47	<0.005	<0.010	0.005	1.034	0.014	0.670	0.300	0.059	Bal.
E12	0.35	0.33	1.02	<0.01	<0.010	0.005	0.952	0.015	0.666	0.301	0.061	Bal.
E13	0.36	0.33	0.49	0.006	<0.010	0.283	0.003	0.015	0.679	0.237	0.061	Bal.
E14	0.36	0.33	0.98	0.009	<0.010	0.271	<0.010	0.016	0.675	0.241	0.062	Bal.
E15	0.37	0.33	0.49	0.458	0.389	0.027	0.003	0.018	0.668	0.240	0.059	Bal.
E16	0.35	0.33	0.98	0.472	0.397	0.005	0.003	0.017	0.661	0.239	0.061	Bal.
E17	0.36	0.33	0.51	0.019	<0.010	0.004	<0.010	0.204	0.664	0.234	0.059	Bal.
E18	0.35	0.32	0.49	<0.005	<0.010	0.003	<0.010	0.413	0.662	0.243	0.058	Bal.
E19	0.38	0.33	0.51	<0.005	<0.010	0.003	<0.010	0.595	0.672	0.233	0.058	Bal.
E20	0.36	0.33	0.45	<0.005	<0.010	0.003	<0.010	0.996	0.661	0.236	0.058	Bal.
E21	0.38	0.34	1.00	0.005	<0.010	0.201	<0.010	0.026	0.678	0.242	0.063	Bal.
E22	0.35	0.33	0.49	0.481	<0.010	0.015	0.004	0.401	0.673	0.241	0.059	Bal.

**Table 6:** Measured contents of alloying elements (wt.%) in the individual phases of the experimental alloys designated E1–E22, including the matrix (M), boride phases (B), and, in some samples, carbide phases (C), as determined by SEM-EDS analysis.

Sample	Chemical Composition	SL	Cr	V	W	Ti	Nb	Mo	Mn	Si	Fe
E1	Fe_B04_Cr04_C035	M	0.62	0	0	0	0	0	0.81	0.52	98.05
		B	1.14	0	0	0	0	0	0.73	0.44	97.69
E2	Fe_B04_Cr1_C035	M	1.14	0	0	0	0	0	0.71	0.43	97.72
		B	1.25	0	0	0	0	0	0.64	0.29	97.82
E3	Fe_B04_V05_C035	M	0	0.4	0	0	0	0	0.72	0.28	98.5
		B	0	1.42	0	0	0	0	0.89	0.11	97.6
E4	Fe_B04_Cr1_V05_C035	M	0.97	0.45	0	0	0	0	0.73	0.33	97.5
		B	1.71	1.74	0	0	0	0	0.94	0.16	95.3
		C	1.5	7.08	0	0	0	0	0.89	0.43	90.1
E5	Fe_B04_Cr04_W05_C035	M	0.47	0	0.46	0	0	0	0.71	0.24	98.1
		B	0.76	0	1.89	0	0	0	0.89	0.62	95.8
E6	Fe_B04_Cr1_W05_C035	M	1.12	0	0.60	0	0	0	0.78	0.32	97.1
		B	1.88	0	1.85	0	0	0	1.75	0.22	94.3
E7	Fe_B04_Cr04_W05_C035	M	0.61	0	0.63	0	0	0	0.63	0.34	97.7
		B	1.15	0	2.52	0	0	0	0.98	0.08	95.3
E8	Fe_B04_Cr1_W05_C035	M	1.08	0	0.54	0	0	0	0.71	0.40	97.3
		B	1.94	0	2.20	0	0	0	0.88	0.03	94.9
E9	Fe_B04_Cr04_Mo08_C035	M	0.47	0	0	0	0	0.47	0.70	0.24	98.1
		B	0.80	0	0	0	0	3.32	0.89	0.15	95.5
E10	Fe_B04_Cr1_Mo08_C035	M	0.99	0	0	0	0	0.48	0.73	0.30	97.5
		B	1.68	0	0	0	0	3.71	0.85	0.22	93.5
E11	Fe_B04_Cr04_Nb1_C035	M	0.5	0	0	0	0.1	0	0.69	0.32	98.3
		B	0.90	0	0	0	0.78	0	0.84	0.15	97.3
		C	0.49	0	0	0	17.6	0	0.69	0.32	80.9
E12	Fe_B04_Cr1_Nb1_C035	M	1	0	0	0	0.09	0	0.74	0.31	97.8
		B	1.98	0	0	0	0.66	0	0.85	0.12	96.3
		C	1	0	0	0	28.7	0	0.6	0.25	69.4
E13	Fe_B04_Cr04_Ti03_C035	M	0.51	0	0	0.09	0	0	0.72	0.26	98.4
		B	0.81	0	0	0.37	0	0	1.07	0.19	97.5
E14	Fe_B04_Cr1_Ti03_C035	M	0.98	0	0	0.09	0	0	0.71	0.27	97.9
		B	1.64	0	0	0.46	0	0	1.05	0.19	96.6
		C	1.13	0	0	15.4	0	0	0.6	0.22	82.6
E15	Fe_B04_Cr04_V05_W05_C035	M	0.54	0.37	0.37	0	0	0	0.8	0.26	97.6
		B	0.75	0.87	0.80	0	0	0	0.86	0.15	96.5
E16	Fe_B04_Cr1_V05_W05_C035	M	1.00	0.40	0.61	0	0	0	0.79	0.29	96.9
		B	1.71	1.39	1.26	0	0	0	0.84	0.09	94.2
E17	Fe_B04_Cr04_Mo02_C035	M	0.54	0	0	0	0	0.15	0.74	0.29	98.3
		B	0.91	0	0	0	0	0.90	0.87	0.20	97.05
E18	Fe_B04_Cr04_Mo04_C035	M	0.52	0	0	0	0	0.78	0.72	0.25	97.7
		B	0.82	0	0	0	0	2.22	0.90	0.23	95.8
E19	Fe_B04_Cr04_Mo06_C035	M	0.56	0	0	0	0	0.45	0.75	0.28	97.9
		B	0.87	0	0	0	0	2.23	0.90	0.25	95.7
E20	Fe_B04_Cr04_Mo1_C035	M	0.55	0	0	0	0	1.53	0.77	0.37	96.7
		B	0.75	0	0	0	0	3.25	0.85	0.29	94.8
E21	Fe_B04_Cr1_Ti02_C035	M	1.02	0	0	0.07	0	0	0.77	0.28	97.8
		B	1.70	0	0	0.39	0	0	1.11	0.55	96.5
		C	1.47	0	0	16.2	0	0	0.69	0.20	81.4
E22	Fe_B04_Cr04_V05_Mo05_C035	M	0.49	0.41	0	0	0	0.38	0.67	0.35	97.7
		B	0.90	1.37	0	0	0	1.82	0.92	0.24	94.7

**Table 7:** Summary statistics and linear regression analysis of the relationship between cell area and distance from the core for individual samples.

Sample	Valid N	Mean (µm^2^)	Median (µm^2^)	SD (µm^2^)	Overall Slope (×10^−2^)	Overall *R*^2^	Slope of Means (×10^−2^)	*R*^2^ of Means	Slope *p*-Value	Theil–Sen Slope (×10^−2^)	OLS–Robust Difference (×10^−2^)
E1	378	183	180	24	−0.748	0.276	−0.730	0.864	<0.001	−0.715	−0.015
E2	398	213	203	49	−1.010	0.144	−1.119	0.823	<0.001	−0.754	−0.365
E3	451	236	224	44	−3.080	0.116	−2.807	0.331	0.064	−5.902	3.094
E4	563	257	251	54	−1.570	0.322	−1.558	0.894	<0.001	−2.196	0.638
E5	409	279	271	41	−1.160	0.23	−0.960	0.692	<0.001	−1.030	0.070
E6	448	250	239	43	−0.826	0.118	−0.791	0.275	0.010	−1.752	0.960
E7	449	233	230	26	−0.768	0.302	−0.815	0.762	<0.001	−1.297	0.482
E8	459	272	263	57	−1.850	0.319	−1.468	0.743	<0.001	−1.874	0.405
E9	619	248	246	29	−1.540	0.783	−1.473	0.953	<0.001	−1.370	−0.103
E10	633	258	249	46	−1.590	0.444	−1.547	0.855	<0.001	−2.058	0.511
E11	628	284	277	28	−1.050	0.426	−0.952	0.744	<0.001	−1.508	0.555
E12	637	303	300	33	−0.994	0.298	−0.974	0.668	<0.001	−1.492	0.518
E13	644	329	338	50	−1.600	0.377	−1.578	0.742	<0.001	−0.859	−0.719
E14	744	292	299	46	−0.832	0.129	−0.679	0.287	0.059	0.056	−0.735
E15	702	341	349	37	−1.080	0.299	−1.265	0.637	0.002	−0.183	−1.082
E16	638	271	268	28	−1.050	0.434	−1.034	0.876	<0.001	−1.203	0.170
E17	700	312	304	51	−1.170	0.222	−1.112	0.719	<0.001	−1.377	0.265
E18	801	274	270	33	−0.671	0.161	−0.675	0.832	<0.001	−0.452	−0.224
E19	641	252	249	24	−0.920	0.451	−0.927	0.809	<0.001	−1.009	0.082
E20	584	259	253	22	−0.999	0.479	−0.941	0.796	<0.001	−0.937	−0.004
E21	597	312	320	31	−1.520	0.658	−1.544	0.915	<0.001	−0.737	−0.807
E22	662	273	275	39	−0.659	0.088	−0.630	0.407	0.035	1.334	−1.964

**Table 8:** Estimated main effects of alloying elements on cell area and radial cell area gradient descriptors. Effects, standard errors, t-statistics, and *p*-values are reported for Cr, V, W, Ti, Nb, and Mo. Model coefficients of determination were *R*^2^ = 0.482 (adjusted R^2^ = 0.275) for cell area, *R*^2^ = 0.187 (adjusted *R*^2^ = 0.000) for mean OLS slope, and *R*^2^ = 0.228 (adjusted R^2^ = 0.000) for the Theil–Sen slope <0.001.

Response Variable	Factor	Effect	Std. Err.	t(15)	*p*
Cell area	Mean/Interc.	365.685	31.612	11.658	<0.001
	(1) Cr (L)	−5.152	25.443	−0.203	0.842
	(2) V (L)	31.711	19.570	1.620	0.126
	(3) W (L)	40.557	22.116	1.833	0.086
	(4) Ti (L)	94.808	27.222	3.482	0.003
	(5) Nb (L)	68.442	28.990	2.360	0.032
	(6) Mo (L)	42.131	28.835	1.461	0.164
Mean slope	Mean/Interc.	−1.20 × 10^−2^	0.003	−3.452	0.004
	(1) Cr (L)	−4.09 × 10^−3^	0.003	−1.461	0.165
	(2) V (L)	−9.97 × 10^−4^	0.002	−0.463	0.650
	(3) W (L)	−2.63 × 10^−5^	0.002	−0.011	0.992
	(4) Ti (L)	−2.48 × 10^−3^	0.003	−0.830	0.420
	(5) Nb (L)	3.38 × 10^−4^	0.003	0.106	0.917
	(6) Mo (L)	−2.42 × 10^−3^	0.003	−0.764	0.457
Theil–Sen slope estimator	Mean/Interc.	−5.50 × 10^−3^	0.008	−0.676	0.510
	(1) Cr (L)	−5.90 × 10^−3^	0.007	−0.900	0.382
	(2) V (L)	3.93 × 10^−3^	0.005	0.780	0.447
	(3) W (L)	−6.23 × 10^−4^	0.006	−0.109	0.914
	(4) Ti (L)	9.70 × 10^−3^	0.007	1.384	0.187
	(5) Nb (L)	−2.29 × 10^−3^	0.007	−0.307	0.763
	(6) Mo (L)	2.14 × 10^−3^	0.007	0.288	0.777

**Table 9:** Sample allocation and mean cell area for groups defined by the principal alloying element.

Alloying Group	Samples	*n*	Mean Cell Area (µm^2^)
Base	E1, E2	2	198
V	E3, E4	2	247
W	E5–E8	4	259
Mo	E9, E10, E17–E20	6	267
Nb	E11, E12	2	294
Ti	E13, E14, E21	3	311

**Table 10:** One-way ANOVA and Kruskal–Wallis comparisons of cell area and regression-derived parameters among alloying groups.

Parameter	ANOVA *F*	ANOVA *p*	Kruskal–Wallis *H*	Kruskal–Wallis *p*
Mean cell area (µm^2^)	8.048	0.001	12.634	0.0271
Median cell area (µm^2^)	11.149	<0.001	12.634	0.0271
|Mean regression slope|	0.324	0.889	1.029	0.960
Mean *R*^2^	0.834	0.548	5.689	0.338

**Table 11:** Statistically significant pairwise differences in mean and median cell area among alloying groups identified by Tukey’s post hoc test.

Parameter	Group 1	Group 2	Difference (µm^2^)	Tukey *p*-Value
Mean cell area	Base	W	−61	0.048
Mean cell area	Base	Mo	−69	0.014
Mean cell area	Base	Nb	−96	0.006
Mean cell area	Base	Ti	−113	<0.001
Mean cell area	V	Ti	−64	0.047
Median cell area	Base	W	−59	0.044
Median cell area	Base	Mo	−70	0.009
Median cell area	Base	Nb	−97	0.004
Median cell area	Base	Ti	−127	<0.001
Median cell area	V	Ti	−81	0.007
Median cell area	W	Ti	−68	0.007

**Table 12:** Summary of boride morphology and network connectivity in the experimental alloys E1–E22.

Sample	Predominant Boride Morphology	Connectivity	Carbides/Another Dark Phase	Characteristic
E1	network-like/irregular	medium	not detected	irregular boride network
E2	network-like/polymorphic	medium	not detected	more segmented network, local branching
E3	fragmented/polymorphic	low–medium	not detected	more fragmented borides
E4	network-like + rod-like	medium	not detected	locally pronounced elongated features
E5	fragmented/polymorphic	low–medium	yes	pronounced disruption of the continuous network accompanied by a carbide phase
E6	fragmented + polymorphic	low	not detected	pronounced dark, more massive features; boride network suppressed
E7	fragmented/fine network-like	low	not detected	fine, predominantly disconnected borides
E8	fragmented/polymorphic	low	not detected	weakly interconnected boride phase
E9	rod-like + fragmented	low	not detected	more pronounced elongated borides
E10	network-like/intergranular	medium–high	not detected	relatively well-developed network
E11	network-like + fishbone-like	medium–high	yes	pronounced branched/eutectic features accompanied by a carbide phase
E12	network-like + polymorphic	medium	yes	borides accompanied by a dark carbide phase
E13	network-like	medium	not detected	relatively regular intergranular network
E14	network-like/fragmented	medium	not detected	finer and partially discontinuous network
E15	fragmented/polymorphic	low–medium	not detected	relatively fine borides
E16	fragmented/fine network-like	low	not detected	markedly limited connectivity
E17	polymorphic/fragmented	low–medium	not detected	locally segmented boride features
E18	polymorphic + fragmented	low–medium	not detected	more heterogeneous morphology
E19	network-like/polymorphic	medium	not detected	locally coarser regions
E20	fragmented/polymorphic	low	not detected	strongly fragmented boride network
E21	fragmented + rod-like	low	yes	isolated segments, locally elongated features
E22	network-like + polymorphic	medium	not detected	partially interconnected network, locally fishbone-like

## Data Availability

The raw data generated and analysed during the current study are openly available in Zenodo at https://doi.org/10.5281/zenodo.23187906. Due to their large file size, the full-resolution microscopy images are stored in the data repository of the Faculty of Engineering, Czech University of Life Sciences Prague, and are available from the corresponding author upon reasonable request.

## Abbreviations

The following abbreviations are used in this manuscript:

ANOVA	Analysis of variance
B	Boron; boride-rich region (phase label in SEM–EDS tables)
Bal.	Balance
C	Carbon; carbide-rich region (phase label in SEM–EDS tables)
CCT	Continuous cooling transformation
DoE	Design of Experiments
EDS	Energy-dispersive X-ray spectroscopy
*F*	ANOVA F-statistic
H	Kruskal–Wallis test statistic
LC	Low carbon (in FeCr LC)
LOD	Limit of detection
M	Matrix (phase label in SEM–EDS tables)
N/*n*	Number of observations/sample size
OES	Optical emission spectroscopy
OLS	Ordinary least squares
*p*	*p*-value
*q*	Multiple-testing-adjusted *p*-value (Benjamini–Hochberg procedure)
*r*	Pearson correlation coefficient
*R*^2^	Coefficient of determination
SD	Standard deviation
SEM	Scanning electron microscopy
t(15)	t-Statistic with 15 degrees of freedom
TTT	Time–temperature–transformation
wt.%	Weight percent
*β*	Regression coefficient (standardised where specified)
*ρ*	Spearman rank correlation coefficient
